# Executive Function from Observation and Reflection Tool (EFFORT): Validation of a Culturally Adaptable and Publicly Available Item Bank in Seven Countries

**DOI:** 10.3390/bs16050693

**Published:** 2026-04-30

**Authors:** Jelena Obradović, Ishita Ahmed, Mateus Mazzaferro, Michael J. Sulik, Dana C. McCoy, Sharon Wolf, Catherine E. Draper, Nikhit D’Sa, Steven J. Howard, Sebastián Lipina, Kavindya Thennakoon, Erfan Ghalibaf

**Affiliations:** 1 Graduate School of Education, Stanford University, Stanford, CA 94305, USA; iahmed2@stanford.edu (I.A.); mazzafe@stanford.edu (M.M.); mjsulik@stanford.edu (M.J.S.); kavindya@stanford.edu (K.T.); 2 Graduate School of Education, Harvard University, Cambridge, MA 02138, USA; dana_mccoy@gse.harvard.edu; 3 Graduate School of Education, University of Pennsylvania, Philadelphia, PA 19104, USA; wolfs@upenn.edu; 4 SAMRC Developmental Pathways for Health Research Unit, University of the Witwatersrand, Johannesburg 2050, South Africa; catherine.draper@wits.ac.za; 5 Global Center for the Development of the Whole Child, University of Notre Dame, Notre Dame, IN 46556, USA; ndsa@nd.edu; 6 Department of Education, University of Oxford, Oxford OX1 2JD, UK; steven.howard@education.ox.ac.uk; 7 Centro de Educación Médica e Investigación Clínica “Norberto Quirno” (CEMIC), Unidad de Neurobiología Aplicada (UNA), Buenos Aires C1431FWO, Argentina; slipina@cemic.edu.ar; 8 Département d’Anatomie, Université du Québec à Trois-Rivières, Trois-Rivières, QC G9A 5H7, Canada; erfan.ghalibaf.kh@gmail.com

**Keywords:** executive functions, assessment, cultural relevance, ecological validity, global settings, young children, adult report

## Abstract

Existing adult-report survey measures provide crucial information about children’s executive function (EF) development across contexts, but lack cultural relevance and ecological validity. To address these limitations, we introduce the Executive Function from Observation and Reflection Tool (EFFORT), a publicly available, open-source item bank designed for cross-cultural adaptation that includes 32 parallel items for caregivers and teachers across six EF domains: sustained attention, response inhibition, interference suppression, working memory, cognitive flexibility, and planning/organization. EFFORT additionally includes 10 assessor report items intended for use following a structured, standardized assessment session. This study presents the first validation of the tool within seven countries (Argentina, Australia, Bangladesh, Haiti, South Africa, Sri Lanka, United States) leveraging caregiver, teacher, and assessor observations of 1738 children (aged 3–11 years). Findings revealed acceptable fit for a six-factor structure for caregiver and teacher reports that were not empirically distinct, but yielded highly reliable composites. We further validated a 12-item short form for caregivers and teachers that demonstrated strong unidimensionality, gender invariance, and age-related increases. We demonstrated significant convergence of a short-form caregiver and teacher composite with the assessor-reported measures, as well as convergence of all three adult reports with direct assessments of children’s EF skills. This new tool holds promise to advance the science of how children develop and apply EFs to accomplish everyday goals across different cultural settings and in understudied populations.

## 1. Introduction

Executive Functions (EFs) are higher-order cognitive skills that support goal-directed behaviors by enabling individuals to stay focused amidst distractions (i.e., inhibitory control—interference suppression), control impulsive or habitual responses (i.e., inhibitory control—response inhibition), manipulate and update information in their minds (i.e., working memory), and flexibly shift between environmental or task demands (i.e., cognitive flexibility). While EFs may be universally relevant for learning and adaptation (Diamond, 2013; Obradović & Willoughby, 2019), EF assessments reflect cultural norms, values, and experiences. Standardized, de-contextualized EF tasks have been criticized for lacking ecological validity and privileging certain cultural assumptions in their design and administration (Jukes et al., 2024; McCoy, 2019; Miller-Cotto et al., 2022). In contrast, adult survey reports of children’s observable behaviors that utilize EFs in everyday settings can capture contextually relevant functioning.

Existing survey assessments of EF behaviors have been primarily developed and tested in North America and Western Europe, and are limited with regard to their cultural representation, conceptual coverage, and adaptation and translation permissions. Given the broad interest in assessing children’s EFs around the world, including in understudied low- and middle-income country (LMIC) settings, the current study is designed to address these limitations by introducing a publicly available item bank that can be used at no cost and adapted for different contexts by adding culturally relevant examples of tasks and situations that elicit EFs. We further provide validity evidence for this new item bank using data on children’s EF behaviors collected from caregivers, teachers, and assessors in Argentina, Australia, Bangladesh, Haiti, South Africa, Sri Lanka, and the United States.

### 1.1. EF Behaviors: What Are They and Why Do They Matter?

EF behaviors can be observed during everyday situations by adults who interact with children and support their development, socialization, and learning. We use the term EF behaviors to reference behaviors that adults can observe and rate and the term EF skills to reference cognitive skills captured using direct assessment tasks. EF behaviors overlap with self-regulation (controlling attention, thoughts, emotions, and actions), but emphasize behaviors in pursuit of specific goals and do not evoke social interactions (McClelland & Cameron, 2012). The focus of this study was to advance our ability to study EF behaviors as a complementary construct to latent EF skills, especially since it has been shown that EF skills and EF behaviors are related yet distinct constructs that share only modest convergent validity in Western, high-income country (HIC) studies (Toplak et al., 2013). To guide this measurement development project, we started with the classic tripartite model of EF that differentiates three foundational domains—inhibitory control, working memory, and cognitive flexibility (Diamond, 2013; Miyake et al., 2000). We extended this model to further divide these domains, and to incorporate behaviors that are EF-adjacent or integrative. Namely, we focus on six domains of EF behaviors that primary caregivers and teachers have regular opportunities to observe in home and classroom contexts.

First, *sustained attention and engagement* are foundational EF-adjacent behaviors essential for accomplishing goals (Garon et al., 2008). While some forms of attention and engagement may not be effortful and do not necessitate EF (e.g., watching a rewarding TV show in a quiet room), other situations require more cognitive effort to sustain attention because of external distractions or lack of intrinsic motivation (e.g., paying attention to a parent explaining a chore or a teacher demonstrating how to complete a group activity often occurs amidst the noises and hubbub of family/classroom interactions). Similarly, when children need to focus on and engage in lengthy, challenging, or repetitive activities, they may use their EFs to resist internal distractions (e.g., feelings of boredom, mind wandering) and persist on such tasks.

The second and third domains focus on different elements of inhibitory control. Namely, the second domain includes *inhibitory control interference suppression* behaviors that reflect children’s efforts to actively ignore irrelevant, interfering, or distracting information or events, such as a noisy sibling, a disruptive classroom visitor, or an opportunity to abandon a required task to do something fun. In turn, the third domain includes *inhibitory control response inhibition* behaviors that showcase children’s abilities to overcome or stop an impulsive, habitual, or overlearned response. While specific behaviors and activities may vary across cultures and contexts, a universal goal of child rearing is to teach children to wait their turn, to stop undesirable behaviors, and, when needed, to disengage from a preferred activity (Bornstein, 2009; Lansford, 2022). Before these behaviors become habitual or automatic responses, children effortfully leverage inhibitory control to learn them. In Western HIC contexts, for example, young children are socialized to follow the rules of conduct by inhibiting their impulses to grab desired food or toys, to wander around when asked to sit still, to skip to the front of a line, to push or hit when upset, or to talk quietly in certain settings. Inhibitory control is also needed to overcome habits when they are no longer appropriate or effective anymore, such as sucking a thumb to calm down or using one’s fingers for more advanced numerical operations.

The final three domains include more advanced EF behaviors that are recruited in situations where children are learning, solving problems, or engaging in cognitively demanding tasks. *Working memory* helps children to mentally update and manipulate information, such as doing numerical calculations in one’s head during classroom math activities or while shopping and preparing ingredients for cooking. Further, working memory supports the successful completion of activities that require completing multiple steps or following lengthy multi-step instructions (e.g., fetching an array of products in a store, gathering materials needed for a classroom activity, preparing a meal, or learning a dance routine). Children also engage working memory when telling a story that includes sequenced events, explaining instructions in an ordered fashion, following multi-step walking directions, or considering and weighing different options when making a choice.

*Cognitive flexibility* may be required when children need to think of a new solution to a problem, repurpose materials or objects in a novel way, understand a conflicting perspective, or adapt behavior to new situational demands. In children, these behaviors can be observed during pretend or role play, negotiations with others, or behavioral adaptations when family plans or classroom schedules change. Finally, many activities that require a certain degree of *planning and organization* may leverage all EF domains. As a result, EF behaviors can also be observed in a child’s preparedness for an activity, strategic and reflective engagement, and time management.

A growing body of research demonstrates the relevance of EFs for school outcomes in children from LMIC settings (Ezeugwu et al., 2025; Willoughby et al., 2019; S. Wolf & McCoy, 2019), corroborating the extensive findings that link EFs with school readiness, adaptive classroom behaviors, and academic achievement in HIC studies (Cortés Pascual et al., 2019; Duncan et al., 2007). However, the majority of these studies have been conducted using traditional direct assessments of EF skills on a specific task not typical in children’s daily lives (Ezeugwu et al., 2025; McHenry et al., 2023), limiting our understanding of the unique role that EF behaviors may have for contextual adaptation and learning.

A few recent LMIC studies suggest that EF skills and EF behaviors may have complementary value in predicting school outcomes. For example, a large study of four-year-olds living in rural Pakistan revealed that both direct assessments of EF skills and adult reports of EF behaviors predicted children’s school readiness (Obradović et al., 2022). Another study of primary school children in Ghana demonstrated that both teacher and assessor reports of EF behaviors predicted children’s scores on literacy and numeracy tests over and above significant contributions of direct assessment of EF skills and previous academic performance (Ahmed et al., 2022). Most of this research employs survey measures developed for HIC settings, sometimes using ad hoc items from surveys not designed to measure EF behaviors per se, or that confound informant and method variance (i.e., differences between reporters could be attributed to differences in survey tools).

Having a standardized, culturally relevant, and ecologically valid approach to assessing EF behaviors across home and classroom settings would facilitate research in understudied settings around the globe to advance our understanding of how EF behaviors develop and how those behaviors are applied by children in their everyday lives. Our understanding of EF development is largely based on a minority of the world’s child population and contexts, which risks missing or misrepresenting crucial insights into EF and its growth. A contextually grounded assessment may help us understand how EF behaviors are related to children’s daily competencies beyond school outcomes. For example, the ability to successfully help with chores (e.g., cooking, shopping, herding animals) or participate in cultural or religious practices (e.g., dancing, weaving/beading, praying) also requires the application of inhibitory control, working memory, and cognitive flexibility.

### 1.2. EF Behaviors: Limitations of Existing Survey Assessments

Widely used survey assessments of EF behaviors originated in HICs typically assume specific socialization practices (e.g., adult-scaffolded engagement with toys and books) and experiences (e.g., widespread attendance in early childhood care and education programs; formal schooling). These surveys may fail to capture the ways that EFs are expressed in LMIC settings. For example, parent- and teacher-reported versions of the Children’s Behavioral Questionnaire (CBQ; Rothbart et al., 2001) include subscales that assess children’s attention focusing, attention shifting, and inhibitory control behaviors. While these subscales show good psychometric properties and predictive validity for social and learning outcomes in studies of children in the United States (Eisenberg et al., 2004; Teglasi et al., 2015), many items presume certain child-rearing experiences and opportunities not universally shared by children around the world (e.g., playing specific games, preparing for trips and outings, watching TV, drawing/coloring, crossing streets, sitting still; e.g., Hermida et al., 2025). Similarly, a more recent measure specifically designed to assess preschoolers’ EF behaviors—Ratings of Everyday Executive Functioning (REEF; Nilsen et al., 2017)—asks caregivers to rate a set of EF behaviors that children may display in specific settings (e.g., while playing games, during story time, around the house, out shopping) that may incorrectly assume familiarity and exposure to culturally specific activities and practices. Further, existing behavioral survey measures like the CBQ primarily include items that assess inhibitory control behaviors that are easier to observe relative to behaviors that leverage working memory and cognitive flexibility. Yet, behavioral applications of working memory and cognitive flexibility become more developmentally salient as children transition to middle childhood and gain more learning opportunities and goal-directed responsibilities.

Other survey measures are also limited by assessing children’s EF difficulties and deficits rather than strengths. This is particularly true of instruments developed to study EF behavioral difficulties as correlates of externalizing behaviors, Attention-Deficit/Hyperactivity Disorder (ADHD), and related symptoms in HIC settings (Doyle et al., 2005; Sulik & Obradović, 2017). This work has frequently employed symptom checklists, such as the Child Behavioral Checklist (CBCL; Achenbach, 1991), that have subclinical and clinical thresholds validated with United States samples. Neuropsychological researchers have also used the child adaptations of the Behavior Rating Inventory of Executive Function (BRIEF; Gioia et al., 2000), a survey measure specifically designed to assess children’s EF difficulties that is also normed with United States samples and linked to various developmental disorders (Gioia et al., 2002; Mahone et al., 2002). Aside from being lengthy, the proprietary nature of these surveys makes them prohibitively costly to use in under-resourced settings, and the emphasis on clinical conditions makes it challenging to adapt across different cultures (Betancourt et al., 2009; Thulin et al., 2022).

Recognizing the need to assess EF deficits in children by removing the semantic overlap between EF behavioral items and the diagnostic criteria of ADHD, Thorell and Nyberg (2008) developed the freely available parent- and teacher-reported Childhood Executive Functioning Inventory (CHEXI). The survey consists of four subscales that form two factors (inhibitory control and working memory difficulties) and has been translated into many languages (see chexi.se). Several studies link higher levels of EF difficulties on the CHEXI to lower academic performance in primary school students from China, Hungary, Iran, Kenya, Spain, and Sweden (Amukune & Józsa, 2021; Thorell et al., 2013). However, because the CHEXI has been used primarily to study neuropsychological deficits and externalizing behaviors—particularly ADHD—it may function best as a transdiagnostic indicator of mental health problems (Zelazo, 2020) and may not capture the full range of EF behaviors in healthy community samples. The absence of difficulties does not imply the presence of EF strengths. For example, a child who remembers to fetch an item their caregiver requested may not excel in the ability to remember and fetch multiple items on their own.

EF behaviors have also been studied in educational research using surveys of children’s self-regulation behaviors, as important markers of school readiness and predictors of adaptive classroom behaviors and academic achievement in HIC studies (Finders et al., 2021; McCoy, 2019; Ursache et al., 2012). This research sometimes uses measures that assess a broader set of self-regulatory behaviors, including emotion regulation (e.g., gets overly excited) and motivation (e.g., finds things boring, hard to get going), limiting the generalizability of these findings across cultures and contexts where values, norms, and socialization practices regarding emotion expression and regulation may differ from Western HIC settings. In addition, this type of work sometimes uses subscales or individual items from teacher survey instruments that were not originally designed to capture EFs per se, making it difficult to replicate, compare, and contrast findings across different studies and settings (Palmer et al., 2025). Having parallel forms of caregiver and teacher reports of analogous behaviors in home and classroom settings would advance our understanding of the unique relevance of EF behaviors for learning and adaptation across different settings.

While most adult-reported surveys of children’s EF behaviors have been designed for use by caregivers and teachers, Smith-Donald and colleagues designed an innovative report of children’s EF behaviors that is completed by an assessor who is trained to administer standardized task-based assessments of EF and related skills. Ratings on the Preschool Self-Regulation Assessment-Assessor Report (PSRA-AR; Smith-Donald et al., 2007) provide a unique adult perspective of children’s ability to focus attention, ignore distractions, control impulses, and manage social and emotional demands during a structured testing interaction. Despite a brief and limited observational window, PSRA-AR scores have been shown to uniquely predict children’s school readiness and outcomes in HIC and LMIC settings, even after controlling for direct assessments of EF skills or other reporters’ observations of EF behaviors (Obradović et al., 2022; Willoughby et al., 2019). Others have translated and adapted select PSRA-AR items for administration with older children, showing that the assessor report of EF behaviors uniquely predicts performance on academic tests (Ahmed et al., 2022; Wei et al., 2021). Unfortunately, the selection and adaptation of PSRA-AR items for use with older children or in LMICs have not been standardized.

Assessor reports may be a more standardized measure of children’s behaviors since assessors do not have a previous relationship with the child and their reports are less subject to biases (McCoy, 2019). Given the benefit and cost-effectiveness of adding assessor report to any study that includes direct assessments of children’s cognitive skills, there is a need to develop an assessor report measure that can be used beyond preschool age and across different cultural settings to advance understanding of how and why the assessor perspective matters for the study of EF behaviors.

### 1.3. Executive Function from Observation and Reflection Tool (EFFORT)

To address the limitations of existing measures and to promote research on how EF behaviors develop and relate to learning and adaptation for children ages 3–12 in diverse cultural settings and understudied LMIC contexts, we have developed the Executive Function from Observation and Reflection Tool (EFFORT). The EFFORT item bank consists of 32 items that map onto six conceptualized domains of behaviors: (A) sustained attention; (B) inhibitory control—response inhibition; (C) inhibitory control—interference suppression; (D) working memory; (E) cognitive flexibility; and (F) planning and organization.

We focused this new item bank on the EF behaviors of children who attend pre-primary and primary schools because EFs have been conceptualized as foundational developmental skills and shown to be important markers of school readiness, classroom engagement, and academic achievement (McCoy & Sabol, 2025). While less researched, EF behaviors have been hypothesized to be important predictors of cultural forms of learning (Messer et al., 2025). We also focused on this period because young children lack the metacognitive capacities to reflect on and report on their own EF behaviors, making adult reports crucial at this developmental stage. We hope that EFFORT will enable new inquiries into the unique contribution of EF behaviors to educational success, as well as to other forms of culturally relevant, goal-directed behaviors and learning outcomes. Finally, by having an item bank that extends beyond the early childhood years (e.g., preschool), we hope to facilitate longitudinal research into the development and relevance of EF behaviors across important developmental transitions.

The key innovation of EFFORT is that its items are explicitly designed to be translated, adapted, and used in diverse cultural settings by including item stems that map onto universally relevant EF behaviors. The right for anyone to use, modify, and share the EFFORT item bank is granted by a Creative Commons license. These universal stems are designed to be accompanied by culturally relevant examples of behavioral manifestations, activities, or settings. For example, all around the world, children are asked by adults to use their inhibitory control to stop some undesirable behaviors, but the specific behaviors may vary across cultures and contexts. Similarly, children across diverse cultures may need to use working memory to complete activities that require following lengthy instructions by holding different steps and updating them in their minds as they complete them. In some cultures, children may have opportunities to demonstrate this skill during board games and classroom group projects, while in other cultures, these opportunities may arise from learning how to cook a meal or performing prayer movements. EFFORT also comes with a set of guidelines that explain the essence of each EF behavior represented by an item to help researchers with translations and cultural adaptations.

EFFORT is also designed to represent a comprehensive set of EF behaviors, including cognitive flexibility behaviors, which have been previously omitted from survey measures. The items are all phrased as positive, strength-based statements, emphasizing the development of EF skills rather than difficulties. Relatedly, we have developed response options that acknowledge the development of EF capacities via adult support and scaffolding. Existing assessments typically include response options that focus on how true the EF item is in representing a child’s behavior or on the frequency of a child showing an EF difficulty. In contrast, EFFORT response options include a four-point scale that captures the independence with which a child demonstrates each EF behavior: (1) always on their own, without any support; (2) mostly on their own, needs brief reminders and support; (3) sometimes on their own, needs regular reminders or moderate support; and (4) not able on their own, requires a lot of support.

First, these responses focus on how a child demonstrates EF behavior when the need or opportunity for that EF behavior arises, rather than how frequently a behavioral difficulty emerges. Frequency may vary across EF domains, as everyday children’s activities and settings offer more opportunities to practice inhibitory control skills than cognitive flexibility skills. Second, response options reflect the fact that the application of EFs in everyday situations is not independent of caregivers’ and teachers’ support. The rating scale emphasizes the level of support needed because EF behaviors are frequently scaffolded by adults in everyday life (Fay-Stammbach et al., 2014). Developmentally, EF progresses from an externally supported cognitive control in early childhood to increasingly internalized and self-directed regulation as children get older. This approach recognizes that early development of EFs is dependent on external support and scaffolding, which decrease with age as behavior becomes more internally regulated. The asset-based framing may also reduce social desirability bias in responses. For instance, an adult may find it more socially acceptable to report that a child “needs support” rather than stating that a child “never” exhibits a certain behavior or performs it with “low quality.” Lastly, we selected a four-point response scale that is simpler to understand than some of the current 5- and 7-point scales, but captures more variability at the higher end of the distribution than 3-point scales, which tend to have skewed responses. To help reporters differentiate between the top two options, instructions state that only children who excel at a specific behavior should receive the highest rating.

In addition to creating parallel caregiver and teacher EFFORT item banks, we adapted a smaller set of EFFORT items to be completed by an assessor after interacting with the child during a structured, standardized assessment session. Given the limited context in which assessors observe children’s behaviors, assessor report items focus on children’s abilities to sustain attention, persist with tasks, ignore distractions, and control their behavior according to the situational demands. Although assessor-reported versions were not available for all EFFORT items, all assessor report items do have analogous parent and teacher versions, enabling the study of children’s EF behaviors across different settings and reporters. Similar to the PSRA-AR (Smith-Donald et al., 2007), the response options for assessor-reported EFFORT items include observable behavioral markers to facilitate training and reliability for assessors in completing this assessment.

### 1.4. Current Study

The current study provides the introduction and first empirical validation of the caregiver, teacher, and assessor versions of EFFORT within different cultural settings. Although we posit that the items reflect universally relevant EF behaviors, we do not suggest that all of the assessed behaviors are universally valued, socialized, or promoted equitably, nor that they follow equivalent developmental trajectories. This initial study was guided by three primary goals.

Goal 1: Full Item Bank Examination. We first sought to evaluate: (a) the 32 parallel caregiver and teacher EFFORT items that were developed to capture six conceptualized EFFORT domains (see the list of the six domains above); and (b) 10 assessor EFFORT items targeting a subset of EF behaviors easily observed in a structured assessment setting. To address this goal, we examined item-level data, factor structure, and reliability across caregiver, teacher, and assessor reports. For caregiver and teacher reports, we hypothesized that the items would fit the structure reflecting the six conceptualized domains. For the assessor report, we conducted exploratory analyses to determine the factor structure of these items (see analytic plan for details).

Goal 2: Short-Form Development. Second, we aimed to derive an initial 12-item EFFORT “short form” that equally represents all six domains, captures the most conceptually relevant behaviors, and demonstrates a single latent dimension of EF behavior across sites. This form was developed to illustrate the utility of the broader item bank and to provide researchers with a pragmatic starting place for conducting context-specific studies of overall/general (rather than domain-specific) EF behaviors. We hypothesized that both caregiver and teacher short form versions would fit a single factor and that 12 items would yield a reliable composite representing a global indicator of EF behaviors.

Goal 3: Validity and Convergence. Finally, we evaluated the validity of the short form and assessor composites in a sample of children reflecting different ages and sites. Using the full analytic sample, we examined: (a) gender invariance and gender group differences; (b) associations with child age; and (c) convergence among reporters. In site-specific samples, we examined convergence among reporters and convergent validity in relation to direct assessments of EF skills. This paper does not address measurement invariance across cultural settings due to differences in the sample age range and reporters that were included across sites. While gender invariance and group differences were exploratory aims, we expected to find an age-related increase in children’s ability to independently display EF behaviors across all reporters. We also expected to see a convergence across different reporters and with children’s performance of EF tasks.

## 2. Materials and Methods

### 2.1. Participants and Procedures

The full analytic sample across all sites includes 1738 children (*M* = 6.73, *SD* = 2.20, 48% female). Of these children, 1262 (73%) have caregiver report data primarily from parents, 892 (51%) have teacher report data, 1193 (69%) have assessor report data, and 1243 (72%) have direct assessment data (see Supplementary Materials Table S3 for patterns of completeness across EFFORT components). Data are drawn from convenience samples collected in seven countries, which allows us to evaluate the item bank across a variety of cultural contexts. Supplementary Materials Table S4 presents the study details for each country. All study sites translated and adapted the items to their local context by adapting the examples to be contextually relevant as needed and translating them into the local language (see site specific descriptions below and Supplementary Materials Text S1 for additional details). Some of the sites (Australia, Haiti, South Africa) added EFFORT to ongoing studies by members of an international group of scholars who are experts in designing, adapting, and implementing EF assessments and have experience studying EFs and related behaviors (Global Executive Function Initiative (GEFI); gefi.stanford.edu), whereas other sites (Argentina, Bangladesh, Sri Lanka) were new studies initiated by the international EF research network members to validate the EFFORT item bank. The current study also includes a convenience sample of caregivers from the United States. Although EFFORT was designed to be developmentally appropriate for children ages 3–12 (i.e., pre-primary and primary students), most samples included in the current validation study represent the younger range of this distribution. Specifically, studies in Argentina, Australia, Haiti, and Sri Lanka focused on the early childhood period. Further, study sites varied in the inclusion of different EFFORT reporters and performance-based task assessments of EF skills (see Section 2.3). While this group science approach ensures broad representations of children from diverse cultural contexts, it is limited by a less unified study design.

Argentina: Purposeful sampling was used to select children from public kindergarten schools in the City of Buenos Aires. The schools were selected in collaboration with officials from the Ministry of Education of the Government of the City of Buenos Aires and school district supervisors based on their socioeconomic representation and collaboration capacity. The sample for this study consisted of 93 children ages 4 to 5 years old (*M* = 5.36, *SD* = 0.56, 47% female). Of these children, 49 (53%) have caregiver report data, 88 (95%) have teacher report data, 46 (49%) have assessor report data, and 81 (87%) have direct assessment data. There are 44 (47%) children with both caregiver- and teacher-reported data, 40 (43%) children with both caregiver and direct assessment data, and 66 (71%) children with teacher and direct assessment data. Out of the half of the caregivers who provided their education information, all of them had completed secondary school. Child assessments and caregiver interviews were conducted at schools by five assessors in Rioplatense Spanish, which is spoken in Buenos Aires. The EFFORT items were translated into Rioplatense Spanish through a multi-step, team-based process involving independent translations, iterative group review, and consultation with field assessors to reach the final version. While most examples closely followed the original English version, some examples were added or modified to better reflect daily family life in the local context (e.g., adding references to handwriting practice or family outings). Data collection took place between July and December 2024.

Australia: The EFFORT items were administered as part of a larger study. At baseline, 277 families were recruited from 43 preschools in regional and metropolitan regions of New South Wales, Australia. The sample for this study consists of 275 children (*M* = 4.68, *SD* = 0.51, 43% female). Of these children, 218 (79%) have caregiver report data, 227 (83%) have teacher report data, 261 (95%) have assessor report data, and 269 (98%) have direct assessment data. There are 179 (65%) children with both caregiver- and teacher-reported data, 213 (77%) with both caregiver and direct assessment data, and 224 (81%) with both teacher and direct assessment data. The majority of the sample spoke English as their primary language at home (94.8%) and EFFORT items were administered in English. Children identified as Aboriginal or Torres Strait Islander comprised 6.6% of the sample, in line with population proportions for this age. Out of half of the caregivers who provided their education information, 95% completed secondary school. The EFFORT items were systematically reviewed and adapted for the Australian early childhood education and care sector by a multidisciplinary team. Minor revisions included changes in terminology (e.g., “teacher” to “educator”), removal of less common examples (e.g., assignment with math calculations), and the addition of analogous examples more familiar to local educators (e.g., waiting patiently in line). Child direct assessments were completed in a quiet area of the child’s preschool. Data were collected between September and November 2024.

Bangladesh: Purposeful sampling was used to select 448 diverse households in the Rangpur and Magura Districts of Bangladesh. Four villages were selected from each district. Interviewers surveyed all households in each village to identify households with children in the relevant age range and survey caregivers about their socioeconomic status (SES) and children’s academic competency. The sample for this study consists of 448 children 5 to 11 years old (*M* = 7.97, *SD* = 1.99, 50% female). Of these children, 448 (100%) have caregiver report data, 58 (13%) have teacher report data, 448 (100%) have assessor report data, and 421 (94%) have direct assessment data. There are 58 (13%) children with both caregiver- and teacher-reported data, 421 (94%) with caregiver and direct assessment data, and 54 (12%) with teacher and direct assessment data. All caregivers provided education information and 20% of them completed secondary school. Four trained local interviewers went to each household and administered the EFFORT survey to caregivers and conducted child assessments in Bangla, which is the local language. Interviewers also administered the teacher survey at the teacher’s home. The EFFORT items were translated into Bangla using a collaborative forward- and back-translation process with review by local researchers to ensure clarity, conceptual equivalence, and contextual relevance. Minor adaptations included simplifying language such as using simpler verbs (e.g., “tells” instead of “communicates”) and the substitution of locally familiar examples (e.g., “builds a house with dirt or mud” in place of “builds a fort”). Data collection took place between August and October 2024.

Haiti: The sample for this study comes from a broader program evaluation of a preschool program that was being conducted in the Nord and Artibonite districts in the North of Haiti. The data was collected as part of the post-test data collection in the Nord department in May 2024. The study sample consists of 353 children ages 4 to 8 years old (*M* = 5.29, *SD* = 0.71, 51% female). Of these children, 353 (100%) have teacher-reported data, 338 (96%) have assessor-reported data, and 351 (99%) have direct assessment data. There are 338 (96%) children with both teacher and direct assessment data. Out of the 70% of caregivers who provided their education information, 6% completed secondary school and 55% completed primary school. In each of the 23 schools where post-test data were being collected from children, trained assessors provided the Level-3 (last year before primary school) classroom teacher with a tablet, asking them to complete the EFFORT survey for each child from whom post-test data was being collected for in their classroom. The EFFORT survey was digitized using the Kobo Toolbox platform, with all data securely uploaded and stored on an encrypted server. All children in the sample identified as Haitian and their primary home language was Haitian Kreyol. The EFFORT items were translated into Haitian Kreyol through a multi-step process with professional translation and review by local educators to ensure clarity, relevance, and feasibility. Minor adaptations included small wording changes to better align examples with common preschool routines (e.g., changing “sharing circle” to “morning meetings”, using “build a house” rather than “build a fort”), with final bilingual review confirming conceptual equivalence.

South Africa: The EFFORT pilot was incorporated into an existing study to test cognitive measures with children, adolescents and caregivers in low-income communities in South Africa. The community-based sample was recruited by a local organization via phone calls and WhatsApp within a low-income, urban community in Cape Town through convenience sampling methods. The sample for this study consists of 104 children 4 to 11 years old (*M* = 7.16, *SD* = 2.33; 50% female). Of these children, 104 (100%) have caregiver-reported data, 100 (96%) have assessor-reported data, and 102 (98%) have direct assessment data. There are 102 (98%) children with both caregiver and direct assessment data. Out of the 70% of caregivers who provided their education information, 84% completed secondary school. Data collection took place at a local church and participants were recruited who lived within walking distance. Two trained research assistants fluent in the local language (isiXhosa) conducted recruitment, consenting, and data collection. All EFFORT items were administered in English, following a review by the locally based research team to assess relevance and feasibility. Minor adaptations were made to item examples to ensure contextual relevance (e.g., replacing classroom-based academic tasks with activities such as building blocks, coloring, playing games, or completing household chores). Data collection took place between March and May 2024.

Sri Lanka: Purposeful sampling was used to select two semi-rural schools in the Kurunegala District of Sri Lanka. The district was selected due to its diversity in language (Sinhala and Tamil speaking communities) and religion (Buddhists and Muslims). The two schools were selected through a community-based approach after obtaining permission and recommendations from the local education office in the province. Participants were recruited directly at the school sites, where the school coordinator selected a purposeful sample based on linguistic, ethnic, and gender diversity from each class to meet the sample requirements. The sample for this study consists of 177 children aged 5 to 8 years (*M* = 6.61, *SD* = 0.59; 45% female). Of these children, 155 (88%) have caregiver-reported data and 166 (94%) have teacher-reported data. There are 145 (82%) children who have both caregiver and teacher-reported data. The majority of participating families were Tamil-speaking (*N* = 96), with a smaller group of Sinhala-speaking families (*N* = 51) and bilingual families (*N* = 8). Almost all the caregivers provided their education information and 97% completed secondary school. A team of 10 community interviewers administered the EFFORT survey to caregivers in their primary local language (Tamil or Sinhala) and teachers completed the survey for students in their class in Tamil or Sinhala based on the language of the school. The EFFORT items were translated into Sinhala and Tamil by certified translators and refined through iterative review with local educators, researchers, and assessors to ensure accuracy, readability, and cultural relevance. Most original examples were retained because they reflected commonly observed behaviors in the Sri Lankan context. Data collection was conducted in September 2024.

United States: This was a convenience sample recruited through the prolific.com website. Caregivers completed a longer survey about one target child that included the EFFORT questions (Lightbody et al., 2026). The sample for this study consists of 288 children ages 4 to 11 years old (*M* = 7.92, *SD* = 2.08; 47% female). Of these children, 288 (100%) had caregiver-reported data. All the EFFORT items were presented in English. No translation or adaptation was needed. Data was collected between January and February 2024.

### 2.2. Analytic Plan

Goal 1: Full Item Bank Examination. We started by examining the item-level descriptive statistics to assess variability in children’s behaviors and to identify any extreme distributions. Additionally, we examined item-level correlations with children’s age and gender. Next, we tested the factor structure of caregiver, teacher, and assessor full item banks using both full analytic and site-specific samples. While the full sample included all sites, site-specific analyses were restricted to those meeting minimum sample size requirements (*N* > 150) for factor analysis with high loadings and multiple latent factors (E. J. Wolf et al., 2013). We estimated exploratory factor analysis (EFA; assessor only) and confirmatory factor analysis (CFA; caregiver, teacher, and assessor) models in R using lavaan 0.6–19 with the robust maximum likelihood estimator (MLR). Teacher models used cluster-robust standard errors at the teacher level. Based on widely adopted recommendations from Hu and Bentler (1998), model fit was evaluated using a combination of the comparative fit index (CFI ≥ 0.95), and root mean square error of approximation (RMSEA ≤ 0.06) and Standardized Root Mean Square Residual (SRMR ≤ 0.08). Internal consistency was assessed using Cronbach’s alpha for all resulting composites.

For the caregiver and teacher full forms, we ran the same CFA model testing the hypothesized six-factor structure. We predicted that all items would load positively onto their respective factors. While we anticipated cross-site variability in loading magnitudes, identifying the most parsimonious factor structure for each specific site and reporter was beyond the scope of the current study. Since the 10 assessor items represented a subset of caregiver and teacher items, we did not have a strong a priori hypothesis regarding their structure. Thus, we split the assessor sample in half and ran an EFA on half of the sample to identify the optimal number of factors. Using the other half of the sample, we used CFA to confirm that this factor structure fit the data well. We present results for the full and site-specific samples, though did not formally test for measurement invariance across sites given the heterogeneity of reporters, cultural contexts, and developmental periods.

Goal 2: Short-Form Development. To ensure equal representation of the six domains, we selected two items per domain using a combined data-informed (e.g., removing items that showed cross-loadings, residual covariances, moderate skew, relatively lower loadings) and conceptually guided approach (e.g., keeping items that were conceptually most relevant to the construct and were well understood across sites). For the caregiver and teacher short forms, we predicted that all 12 items would load positively onto a single factor.

Goal 3: Validity and Convergence. First, we examined gender invariance of the short-form composites in the full samples by evaluating configural, metric, and scalar invariance. Configural invariance was based on acceptable model fit in each group (Hu & Bentler, 1999). Metric and scalar invariance were supported if the likelihood ratio test was non-significant and there was a decrease in CFI of 0.005 or less (Chen, 2007). When scalar invariance was supported, we tested for gender differences in caregiver, teacher, and assessor reports. Second, we evaluated age-related associations for each reporter in the full samples and expected to find positive associations for all three reporters. Third, we examined interrater convergence using bivariate correlations within the full and site-specific samples. We expected to find low-to-moderate positive associations across different pairs of reporters. Lastly, we assessed convergent validity within site-specific samples by regressing the EFFORT scores on children’s performance on EF tasks, controlling for child age and gender. Consistent with previous work, we expected to find low-to-moderate positive associations between different reporters and EF task scores.

### 2.3. Measures

Demographics: Each study site collected data on child age and gender. Some sites also collected information on household socioeconomic status (e.g., parent education, household income), ethnicity/race, and contextual information. The analyses in this study primarily use child age and gender as they were available at all sites and are comparable.

EFFORT Survey of EF Behaviors: After a review of existing surveys and discussion of conceptual domains, the lead author and three co-authors (IA, DM, SW) drafted items that captured the six hypothesized domains of EF behavior described above. Each item consisted of a stem that described a type of behavior universally relevant across different settings and culturally relevant examples of behavioral manifestations, activities, or settings. These authors also crafted guidelines to help with translations and cultural adaptations (see Supplementary Materials Text S1).

After review, the authors solicited feedback from the GEFI network members. Each domain was reviewed by a group of scholars, and their feedback was discussed and incorporated. For example, the item focused on ignoring distractions was revised to include the adjective “irrelevant” since, in some contexts, it is adaptive to pay broad attention to surrounding activity/noise that could alert children to potential danger or others’ needs. Another example was expanding the “pays attention” item to include adult demonstrations, as in many cultures, adults are more likely to show children how to do something than to tell them. The final 32 items are reported in Table S1; bold item numbers indicate those selected for the short form.

Direct Assessment of EF Skills: The specific EF tasks employed varied across sites. Inhibitory control was assessed using the Hearts and Flowers task in Argentina, Bangladesh, and South Africa and a Go/No-Go task in Australia. Short-term and working memory were assessed using the visual memory task in Argentina and Bangladesh and digit span tasks in Haiti and South Africa. Cognitive flexibility was assessed using a mixed block of the Hearts and Flowers task in Argentina, Bangladesh and in South Africa. General EF was assessed using the Head Toes Knees Shoulders task (HTKS; McClelland et al., 2014) in Australia and Haiti. We include a description of each task and how composites were created.

*The Hearts and Flowers task* (HF; Davidson et al., 2006) measures inhibitory control and cognitive flexibility. The tablet-based version of this task (part of the Assessment of Motivation, Effort, and Self-Regulation (AMES) battery (Obradović, 2019)) was administered in Argentina and Bangladesh. This version of the HF task consists of three blocks: a block of 16 congruent ‘hearts’ trials, a block of 20 incongruent ‘flowers’ trials, and a block of 20 mixed ‘heart and flower’ trials. For congruent heart trials, children were instructed to press the button on the same side as the presented stimulus (i.e., heart) to build a prepotent response. For incongruent flower trials, children were instructed to press the button on the opposite side of the stimulus (i.e., flower) to measure their response inhibition. The stimuli for the hearts and flowers trials appeared on the screen for 1500 ms. For mixed trials, children were presented with both heart and flower trials to assess their ability to switch between the two rules. The stimuli for the mixed trials appeared on screen for 2000 ms. In South Africa, a different tablet-based version of the HF task was administered (Blumenthal et al., 2026). It consisted of three blocks: a block of 12 congruent ‘hearts’ trials, a block of 12 incongruent ‘flowers’ trials, and a block of 36 mixed ‘heart and flower’ trials. Each block began with four practice trials that included feedback for correct and incorrect responses. Each trial started with a 500 ms fixation, followed by stimulus presentation. The stimuli for all the blocks were displayed for 1500 ms. Across both versions, data was processed the same way. A lack of response on a trial was coded as incorrect. Children who had accuracy lower than 50% on the hearts trials were excluded from the analysis. The analysis variables are created by calculating the proportion of correct responses for the incongruent flowers trials and for the mixed trials.

The *Go/No-Go task* measures inhibitory control and was administered in Australia using the Early Years Toolbox (EYT) on an iPad (Howard & Melhuish, 2017). There are “go” trials where children catch a fish by tapping the screen and “no-go” trials where children avoid sharks by resisting tapping the screen. The “go” trials are presented 80% of the time and “no-go” trials are presented 20% of the time so children build a prepotent response to tap the screen and need to inhibit this when a “no-go” trial is presented. To condition the no-go response accuracy on the strength of the pre-potent response (as indicated by go accuracy), the analysis variable was created as the product of go and no-go accuracy scores (range: 0–1).

*The Memory Game task* measures visuospatial short-term and working memory. Since the task does not rely on familiarity with numbers or words, the task performance is not confounded with children’s literacy or numeracy skills. The tablet-based version of this task (part of the AMES battery; Obradović, 2019) was administered in Argentina and Bangladesh. The task consists of forward and backward blocks. Children viewed a sequence of colored squares that lit up in an unpredictable pattern in a 3 × 3 grid. In the forward block, assessors instructed children to touch the squares in the order in which they lit up. In the backward block, assessors instructed children to touch the squares reversing the order in which they lit up. Similar to other span tasks, the sequences of squares became progressively longer—and thus increasingly difficult. There were three trials for each sequence length. Each block ended after three consecutive incorrect trials. The analyzed variables were created by summing the total number of correct answers for each block.

*The Digit Span task* measures short-term and working memory by assessing children’s ability to repeat numbers in different sequences of increasing length. In the forward block, the assessor read the sequence of numbers and the child was instructed to repeat the numbers in the same order. In the backward block, the assessor read the sequence of numbers and the child was instructed to repeat the numbers in the reverse order. In South Africa, both the forward and backward blocks were administered on a tablet as two separate tasks (Blumenthal et al., 2026). Both were presented with ascending difficulty. The blocks had increasing span lengths. Trials with span lengths of two and three were administered as practice trials and included feedback. The span lengths for the test trials were four to nine digits, with two trials per span length (e.g., two 4-digit trials, two 5-digit trials, etc.). If the child scored incorrectly for two trials at the same span length, the task ended. The tasks also ended after the maximum span length was reached. Each digit was displayed for 1 s. The analysis variables were created by summing the total number of correct answers for each block. In Haiti, researchers used an abbreviated four-item version of this task that is a part of the International Development and Early Learning Assessment (IDELA) tool. This version consists of only the forward block that is administered verbally by an assessor. The first item has two numbers and each item increases by one number, so the fourth item has five numbers. This measures short-term memory and is appropriate for younger children. The analysis variable is the sum of the total number of correct answers.

*Head Toes Knees Shoulders task* (HTKS; McClelland et al., 2014) measures children’s general EF skills because it requires children to inhibit their prepotent response to touch a body part that is different from what the assessor states, use working memory to keep each rule on the body correspondences in mind, and use cognitive flexibility to switch between the different rules. In the first block, if the assessor tells the child to “touch their head,” the child is supposed to “touch their toes” and vice versa. In the second block, an additional body correspondence is introduced where if the assessor tells the child to “touch their knees,” the child is supposed to “touch their shoulders” and vice versa. In the third block, the body correspondences change and children must switch between them. If the assessor tells the child to “touch their head,” then the child is supposed to “touch their knees” and vice versa. Additionally, if the assessor tells the child to “touch their shoulders,” then the child is supposed to “touch their toes” and vice versa. In Australia, researchers administered three blocks of 6 practice trials and 10 test items. The analysis variable is a sum accuracy score for practice and test trials, with a correct response scored as 2 and a self-corrected response scored as 1 (incorrect is 0). In Haiti, researchers used an abbreviated 5-item version of this that is a part of the IDELA tool. This version consists of only the head-toes correspondence items. The analysis variable is average accuracy on 5 items.

## 3. Results

### 3.1. Descriptive Item-Level Statistics

Caregiver and Teacher Reports: Table 1 shows the descriptive statistics (mean, standard deviation, skew) for all caregiver and teacher items in the full sample. Overall, all items show good variability, and most approximated a normal distribution. Nine parent items showed mild-to-moderate negative skew (i.e., skew between −0.5 and −1.0), and none were highly skewed. No teacher items exhibited significant skewness. Item-level histograms for both caregiver and teacher reporters can be found in Supplementary Materials (see Figures S1 and S2). With a few exceptions, the caregiver version of the EFFORT items had higher means compared to the teacher versions of the same items. Across all 32 items, all of which had a possible range of 1 to 4, the average item mean for caregiver reports was 2.94 (*SD* = 0.25) and for teacher reports was 2.75 (*SD* = 0.24). Item-level mean differences were not explicitly tested since they are derived from overlapping but not entirely identical samples.

Table 1 also lists item-level correlations with children’s age and gender in the full sample. Associations with children’s age varied across the 32 items, with all significant correlations showing positive association with age (parent *r* = 0.07–0.44, teacher *r* = 0.03–0.26). Children’s gender was largely unrelated to both parent and teacher item-level reports in the full sample.

Assessor Report: Table 2 shows the descriptive statistics (mean, standard deviation, skew) for the 10 assessor items in the full sample. Overall, items showed reasonable variability, with 3 items showing strong negative skew. Item-level histograms for the assessor reports can be found in the Supplementary Materials (Figure S3). Across 10 items, all of which also had a possible range from 1 to 4, the average mean for assessor-reported items was 3.21 (*SD* = 1.32).

### 3.2. Full Caregiver and Teacher 32-Item Bank

Factor Structure: Table 3 presents the fit indices and factor loadings for the 32-item, six-factor CFA model. In the full sample, the six-factor model yielded acceptable fit for both caregiver reports (*CFI* = 0.923; *RMSEA* = 0.043; SRMR = 0.039) and teacher reports (*CFI* = 0.931; *RMSEA* = 0.050; SRMR = 0.034). In the full sample, all standardized factor loadings were moderate to high (ranging from 0.52 to 0.72 for caregivers and 0.61 to 0.81 for teachers). Furthermore, the correlations among the six factors were very high in the full analytic samples, ranging from 0.80 to 1.00 for caregivers and 0.85 to 0.98 for teachers (see Table S2 in Supplementary Materials). This suggests that the six factors were not independent in caregiver and teacher reports.

Table 3 also shows results for the six-factor CFA model for individual sites with at least 150 observations. With one exception, the fit of this model was acceptable (*CFI* = 0.903–0.934; *RMSEA* = 0.039–0.077; SRMR = 0.034–0.056) and all standardized factor loadings were moderate to high (ranging from 0.44 to 0.77 for caregivers and 0.50 to 0.91 for teachers) across all sites. The model for the parent reports in Sri Lanka was the exception, showing poor model fit (*CFI* = 0.725; *RMSEA* = 0.069; SRMR = 0.082) with standardized factor loadings ranging from 0.31 to 0.72. This stood in contrast to the adequate model fit (CFI = 0.929; RMSEA = 0.095; SRMR = 0.035) and high factor loading (all above 0.78) found for the teacher reports in the Sri Lanka sample.

Reliability: As shown in Table 3, the reliability statistics for each of the six subscale composites were high across both reporters in the full sample (Cronbach’s alphas ranged from 0.78 to 0.88). The reliability statistic for each of the six subscale composites was acceptable across both reporters in the site-specific samples (*α* > 0.70) with the exception of caregiver reports in Sri Lanka (*α* > 0.60).

### 3.3. Assessor 10-Item Bank

Factor Structure: Because we had no previously hypothesized assessor model, we first conducted EFA using half of the full sample. We estimated 1-, 2-, and 3- factor solutions. Fit for the 1-factor model was poor. Fit for the 2-factor model was acceptable (TLI = 0.946, RMSEA = 0.102) and significantly better than the 1-factor model (Δχ^2^(9) = 434.38, *p* < 0.001). Although the 3-factor solution fit better relative to the 2-factor solution (Δχ^2^(8) = 107.52, *p* < 0.001), only one item had a primary loading on the third factor (item 10, loading = 0.47), and cross-loaded heavily on the first factor (loading = 0.42). We decided to proceed with the two-factor model because it was the most parsimonious, best-fitting solution that was conceptually meaningful. To confirm this factor structure, we estimated a 2-factor CFA model in the second half of the data, which demonstrated good fit (CFI = 0.973; RMSEA = 0.061; SRMR = 0.028). Next, we evaluated this model using CFA with both the full sample and site-specific samples.

Table 4 presents the fit indices and factor loadings for the 10-item, two-factor CFA model. In the full sample, this model yielded a good fit (*CFI* = 0.968; *RMSEA* = 0.067; SRMR = 0.026). The first factor comprised items reflecting *sustained attention*, with the standardized factor loadings ranging from 0.72 to 0.90. The second factor included items reflecting *behavioral control*, with the standardized factor loadings ranging from 0.77 to 0.88. The two factors were highly correlated (*r* = 0.83).

Table 4 also shows results for the two-factor CFA model for individual sites with at least 100 observations (appropriate N for a two-factor model with high loadings and more than 4 indicators; E. J. Wolf et al., 2013). With one exception, the fit of this model was good across all sites (*CFIs* > 0.950; *RMSEAs* < 0.090, SRMRs < 0.050) and all loadings were moderate to high (ranging from 0.65 to 0.95 for sustained attention and 0.61 to 0.88 for behavioral control). The model in South Africa was the exception, demonstrating relatively lower model fit (*CFI* = 0.890; *RMSEA* = 0.107; SRMR = 0.061), though all standardized factor loadings remained above 0.48.

Reliability: As shown in Table 4, the *sustained attention composite* (six items) showed high reliability in the full sample (*α* = 0.94) and in site-specific samples (*α* range = 0.85–0.95). The behavioral control composite (4 items) also showed good reliability in the full sample (*α* = 0.89) and in the site-specific samples (*α* range = 0.78–0.91).

### 3.4. Short 12-Item Caregiver and Teacher Form

Item Reduction Process: To produce the 12-item short form of the teacher and caregiver EFFORT survey, our goal was to select only two items from each of the six EF domains to ensure broad conceptual coverage in a short format that can be completed quickly. This step ensured that the short form included equal representation of the six domains. We began the reduction process in the full sample by focusing on data quality, first dropping three items (A4: maintains focus in child-led activities; F1: gathers materials before activities; and D6: remembers physical directions) due to moderate skewness and relatively low variability for caregiver reports. Using the remaining 29 items, we estimated a six-factor model for the caregiver and teacher surveys. We then removed six items that showed substantial reductions in model fit due to unmodeled residual covariances or cross-loadings for at least one reporter. These six items were: A3: remains focused and engaged; C3: stops playing when asked; C5: thinks before acting; D4: waits for their turn to act; F2: creates a plan for completing tasks; and F4: checks for mistakes. The resulting six-factor, 23-item model fit well for both caregivers (CFI = 0.956; RMSEA = 0.038; SRMR = 0.030) and teachers (CFI = 0.957; RMSEA = 0.047; SRMR = 0.028). From this pool of items, we selected two items per domain that we judged to be the most salient and complementary indices. The final set of 12 items have bold item numbers in the first column in Table 1.

Factor Structure: To confirm the unidimensionality of our final set of 12 items, we estimated a single-factor CFA model (see Table 5 for fit indices and factor loadings). In the full sample, the one-factor model yielded a good fit for both the caregiver reports (CFI = 0.952; RMSEA = 0.051; SRMR = 0.034) and teacher reports (CFI = 0.973; RMSEA = 0.049; SRMR = 0.026). In the full sample, all standardized factor loadings were average to high (ranging from 0.54 to 0.71 for caregivers and 0.66 to 0.81 for teachers).

Table 5 shows results for the one-factor CFA model for individual sites with at least 150 observations. Across all sites, the fit of this model was acceptable to good (CFI = 0.923–0.946; RMSEA = 0.043–0.105; SRMR = 0.033–0.061). All standardized factor loadings were moderate to high (ranging from 0.30 to 0.82 for caregivers and 0.49 to 0.90 for teachers).

Reliability: As shown in Table 5, the reliability statistics were high for the single composite of 12 items for both caregiver (*α* = 0.88) and teacher (*α* = 0.93) in the full sample. The reliability of the short-form composite was also high across specific sites (caregiver *α* > 0.84; teacher *α* > 0.95).

### 3.5. Associations with Child’s Age and Gender

**Figure 1:**
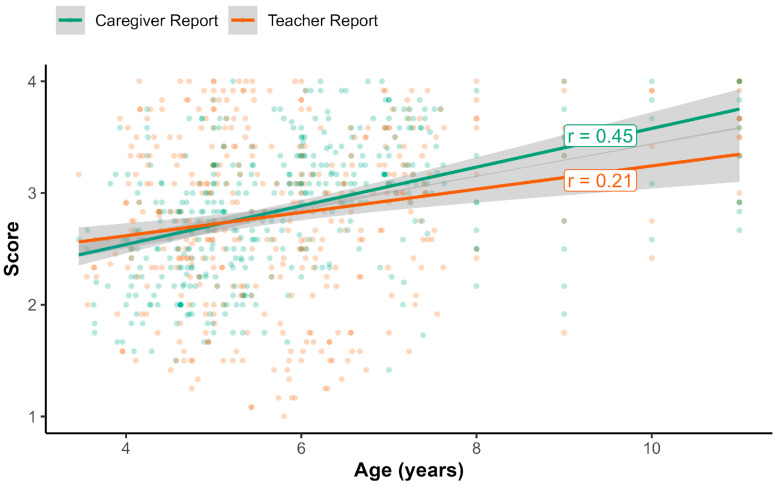
EFFORT 12-item scores by Age in Full Sample.

**Figure 2:**
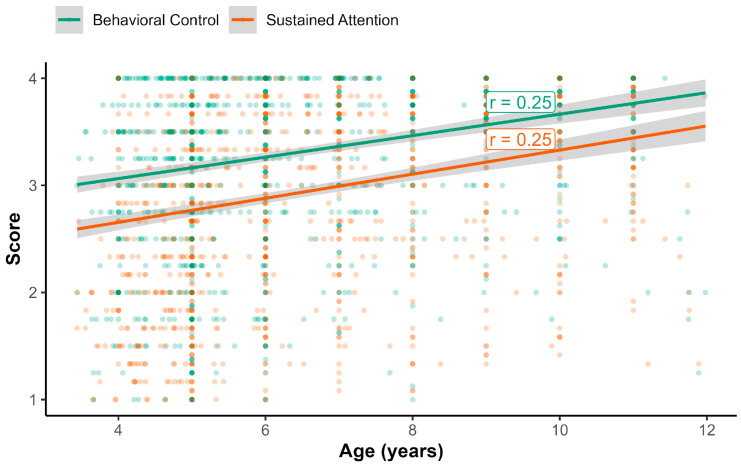
Assessor Scores by Age in Full Sample.

In the full sample, the correlation with age was 0.31 for caregiver reports, 0.20 for teacher reports, and 0.25 for both the assessor-reported attention and behavioral inhibition composite scores (see Figure 1 and Figure 2). For caregiver and teacher reports, we directly compared their dependent correlations with age in the subsample that had both reporters (*n* = 419). Among these participants, the association with age was stronger for caregiver reports (*r* = 0.45) relative to teacher reports (*r* = 0.21, *t*(416) = 4.47, *p* < 0.001).

Measurement invariance across gender was tested using multi-group CFA for the 12-item single-factor short form (Table 6). The configural model showed good fit for teachers (χ^2^ = 218.77, df = 108, *p* < 0.001; CFI = 0.972.; RMSEA = 0.050; SRMR = 0.030), caregivers (χ^2^ = 301.81, df = 108, *p* < 0.001; CFI = 0.952; RMSEA = 0.052; SRMR = 0.037), and assessors (χ^2^ = 273.19, df = 68, *p* < 0.001; CFI = 0.967; RMSEA = 0.068; SRMR = 0.028), indicating that the factor structure was consistent across gender. Imposing equality constraints on factor loadings (metric invariance model) resulted in negligible changes in fit indices (ΔCFI < 0.01; ΔRMSEA < 0.01) for teachers, caregivers, and assessors, supporting metric invariance for each of the EFFORT reports across gender. Imposing constraints on factor intercepts (scalar invariance model) did not significantly alter model fit for teachers, caregivers, or assessors, supporting scalar invariance across gender and allowing for meaningful comparisons of latent means.

Teachers reported slightly better EF behaviors for female children than male children (*r* = 0.12; *t* = −3.32, *p* < 0.001, Cohen’s *d* = −0.23). There were no gender differences in caregiver reports (*t* = −0.16, *p* = 0.872) or in assessor reports of attention and behavior inhibition factors (*p*s > 0.397).

### 3.6. Convergence Across EFFORT Reporters

Within all sites, there was evidence of convergent validity across different reports of children’s EF behaviors with low-to-moderate positive correlations among the composite scores (Table 7). The correlation between teacher reports and caregiver reports was around 0.40 in Argentina, Australia, and Bangladesh. The magnitude of the correlation between teacher and caregiver reports was smaller in Sri Lanka (*r* = 0.26) and in the full sample (*r* = 0.27).

The assessor reports of children’s behaviors showed higher correlations with teacher reports than with caregiver reports in Argentina (*r*s = 0.57 and 0.56 for teacher vs. 0.26 and 0.27 for caregiver) and Australia (*r*s = 0.31 and 0.35 for teacher vs. 0.14 and 0.15 for caregiver). In Bangladesh, the assessor reports showed higher correlations with the caregiver reports than with the teacher reports (*r*s = 0.48 and 0.50 for caregiver and 0.26 and 0.28 for teacher), which may be due to the smaller sample size of teachers in this study. The magnitude of the correlation between assessor reports and caregiver reports was similar in South Africa (*r*s = 0.53 and 0.43) and in the full sample (*r*s = 0.50 and 0.51). There was a smaller correlation of 0.24 and 0.21 between assessor reports and teacher reports in Haiti and 0.26 between assessor reports and caregiver reports in Sri Lanka.

### 3.7. Convergence with Direct Assessments of EF Skills

We also found evidence of convergent validity of the different reports of children’s EF behaviors with direct assessments of EF skills within the sites (Table 8). The caregiver reports were positively associated with (1) HTKS (*β* = 0.21, *p* = 0.001) in Australia; (2) both HF blocks (*β* = 0.15 to 0.17, *p* < 0.001) and Memory Game Forwards block in Bangladesh; (3) the HF Mixed Block (*β* = 0.26, *p* = 0.01) and both the Forward (*β* = 0.17, *p* = 0.04) and the Backward (*β* = 0.28, *p* = 0.01) digit spans in South Africa.

The teacher reports were positively associated with (1) the HF Flowers block (*β* = 0.39, *p* = 0.01), HF Mixed block (*β* = 0.43, *p* = 0.001), and both blocks of the Memory Game (*β* = 0.33, *p* = 0.01 to 0.06) in Argentina; (2) the HTKS (*β* = 0.26, *p* < 0.001) and Go/No-Go (*β* = 0.19, *p* = 0.01) in Australia; (3) the Memory Game Forward block (*β* = 0.28, *p* = 0.02) and Backward block (*β* = 0.26, *p* = 0.03) in Bangladesh; (4) the IDELA HTKS (*β* = 0.21, *p* < 0.001) in Haiti.

The assessor report attention and behavior factors were both positively associated with (1) the HF blocks (*β* = 0.61 to 0.71, *p* < 0.001) and the Memory Game Backwards block (*β* = 0.31, *p* = 0.08 to 0.09) in Argentina; (2) the HTKS (*β* = 0.28 to 0.41, *p* < 0.001) and Go/No-Go (*β* = 0.35 to 0.48, *p* < 0.001) in Australia; (3) the HF blocks (*β* = 0.26 to 0.38, *p* < 0.001) and the Memory Game blocks (*β* = 0.27 to 0.40, *p* < 0.001) in Bangladesh; (4) IDELA HTKS (*β* = 0.10 to 0.21, *p* = 0.00 to 0.06) and IDELA Forward Memory span (*β* = 0.10 to 0.12, *p* = 0.03 to 0.07) in Haiti; (5) the HF blocks (*β* = 0.28 to 0.37, *p* = 0.00 to 0.01) in South Africa. Only the assessor attention factor is positively associated with the Forward Memory span (*β* = 0.31, *p* < 0.001) and only the assessor behavior factor is associated with Backward Memory span (*β* = 0.23, *p* = 0.06) in South Africa.

## 4. Discussion

There is growing interest in studying how EF develops and contributes to children’s competencies globally; however, this necessitates more culturally relevant measures of EFs. Given the limited ecological validity of traditional EF tasks (Hughes, 2023; McCoy, 2019), it is important to develop culturally adaptable and valid survey measures of children’s EF behaviors. In this study, we introduce the new EFFORT item bank, which can be adapted for different contexts by adding culturally relevant examples of tasks and situations. Unlike surveys with a fixed set of questions, an item bank approach enhances measurement validity by minimizing the confounding of individual and cultural differences. Our analyses show that this item bank captures variability and yields reliable indices of caregiver-, teacher-, and assessor-reported EF behaviors that exhibit age-related increases. Furthermore, we demonstrate significant convergence of a short-form composite across different reporters and with direct assessments of EF skills. The item bank adaptation and translation guidelines, together with the validation of a short form, offer new opportunities to advance the science of how EF behaviors support development and learning in traditionally understudied populations.

### 4.1. Variability and Structure of EF Behaviors

Two innovative aspects of the EFFORT item bank include positive, strengths-based item wording and response options that reflect the independence with which a child demonstrates EF behaviors. Examination of item-level data revealed that average caregiver and teacher ratings mostly fell between 2 and 3, corroborating the effectiveness of the survey instructions, which emphasized that “children who excel at a specific behavior should receive 4 (the highest rating), whereas most children will score between 3 and 2.” Limited item-level skewness further demonstrated that the EFFORT items captured significant behavioral variability. This is important given the typical skew of similar surveys and social-desirability pressures inherent in caregiver and teacher reports of young children’s behaviors (e.g., Dekker et al., 2017; Sulik & Obradović, 2018). Further, strength-based phrasing helped capture variability in the presence of an ability beyond the absence of a behavioral problem, a common limitation of symptom checklists. Interestingly, assessor-reported data had higher item means and greater skew, likely due to limited observation windows and the one-to-one assessment context, which tends to promote greater compliance and easier behavioral regulation during a structured interaction.

Across caregiver and teacher reports, the 32-item, six-factor solution yielded acceptable fit, but the six factors were not empirically distinct given high factor correlations in the full samples. The lack of differentiation could be due to the relatively narrow age range of children across the majority of the sites, mirroring prior work that demonstrates a single factor structure of EF skills in young children (Wiebe et al., 2008). With development, EF behaviors, like EF skills, may show more differentiation and studies that include older children may reveal more complex structure of more independent EF strengths. For example, research using CHEXI surveys has revealed a two-factor solution mapping onto inhibitory control and working memory difficulties (Amukune & Józsa, 2021; Camerota et al., 2018; Thorell & Nyberg, 2008). Absolute fit indices suggest further exploration of alternative models. To differentiate domain-general from domain-specific aspects of EF behaviors, one could employ (1) a bifactor model, where a single factor captures general EF variance while separate factors capture domain-specific variance, or (2) a hierarchical model, in which specific EF domain factors load on a higher-order factor. Furthermore, it is essential to investigate the age-related developmental differentiation of EF behaviors within specific cultural context and how schooling experiences influence this process.

This future site-specific work should also examine differences in factor structure across reporters. For example, poor fit was found for the six-factor model with caregiver reports in Sri Lanka, but there was adequate fit for teacher reports. Discrepancies in model fit could stem from caregivers’ limited opportunities to observe certain EF behaviors in a home setting (e.g., young children’s limited engagement in family activities that elicit some EF behaviors, a reporting adult may have limited insights into a child’s behavior due to prevalence of multigenerational caregiving). Across 32- and 12-item caregiver factor solutions, the item “understands conflicting perspectives and ideas” had the lowest loading, corroborating field notes documenting caregivers’ struggles with observing behavioral manifestation of more complex cognitive skills. Understanding conflicting perspectives in family contexts often emerges in the context of interpersonal conflict, which in Sri Lanka children are socialized to avoid (Bolz, 2002; Croucher et al., 2012). However, the adequate fit of a 12-item, single factor solution suggests that caregivers in Sri Lanka perceive a subset of EF behaviors as a single construct. Future studies need to test how the structure of observed EF behaviors changes with age, especially during the transition to formal schooling in contexts where low parental education and a lack of widespread early childhood education programs may affect how EF behaviors are socialized and scaffolded in family settings. Qualitative interviews with reporters could illuminate how EF behavioral manifestations are perceived across home and school settings and whether caregivers and teachers use different references (e.g., siblings, peers) to determine what constitutes optimal or independent behavior in each setting.

Our analysis further revealed that the assessor-reported items reflect two related factors in the full sample. The first factor represents children’s sustained attention, ability to ignore distractions, and tendency to persist and be thorough. The second factor captures children’s ability to behave appropriately by not rushing, waiting to speak, and stopping undesirable behaviors during the assessment session. This solution yielded good fit in Australia, Bangladesh, and Haiti, with South Africa being an exception, possibly due to a much smaller sample size and more heterogeneous age distribution. This finding diverges from previous studies where a subset of PSRA-AR ratings yielded a single attentional and behavioral self-regulation factor (Ahmed et al., 2022), often separate from a factor capturing positive engagement and affect (Obradović et al., 2022; Willoughby et al., 2019). If the two-factor solution is replicated across diverse settings and ages, it could be used to identify different patterns of self-regulation strengths.

Across all factor solutions, the reliability of derived domain composites from the full item bank was high for caregiver, teacher, and assessor reports. Further, a 12-item, single-factor solution also demonstrated acceptable fit across reporters and sites. This indicates that, across diverse settings, the item bank yielded reliable composite measures for the six EF domains and for the single, global EF construct. Items from the EFFORT item bank can, therefore, be selected to study how EF behaviors are shaped by context or relate to other measures of development, learning, and well-being at varying levels of granularity. Researchers seeking a nuanced understanding of how EF behaviors emerge, differentiate, and change should use the full item bank to select or adapt relevant items. For example, a study focusing on multi-method assessments of working memory in Bangladesh employed the working memory items from the full item bank to examine unique predictive validity of different working memory tasks over and above related working memory behaviors for cognitive and religious competencies (Ahmed & Obradović, 2026). In contrast, the short form provides a good starting point for general inquiries into EF behaviors as a unidimensional construct.

### 4.2. Caregiver, Teacher, and Assessor Perspectives

Children’s opportunities to engage EF behaviors vary across family and school settings. At the same time, both caregiver and teacher reports of EF behaviors can reflect biases unique to reporters’ social stereotypes and personal experiences. Studies show that adults’ subjective ratings of EF-related behaviors can be biased by impressions of children’s overall behavior, appearance, or demographic characteristics (e.g., Brandmiller et al., 2020; Fitzpatrick et al., 2016; Ready & Wright, 2011), and may reflect adults’ own well-being (e.g., Joyner et al., 2009; Silver, 2014) or beliefs and identities (Yoder & Williford, 2019). Examination of these biases is critical, as both reporters provide a unique perspective on children’s functioning essential to understanding how to promote healthy development and learning. This work necessitates the use of parallel forms of caregiver and teacher reports to reduce measurement error present in previous work on the unique predictive validity of multiple reporters leveraging different survey instruments (Ahmed et al., 2022). With parallel forms of the caregiver and teacher EFFORT surveys, researchers can interrogate these biases and understand how children’s strengths and areas of growth vary across the two most salient socializing contexts—home and school. Such inquiries can test if reporter congruence varies based on children’s sociodemographics, temperament, or conduct. By employing multiple reporters, researchers can also derive latent measures of EF behaviors to isolate common variance from reporter-specific differences. Additionally, factor analytic approaches can explicitly model differences due to context and perspective (see Kraemer et al., 2003).

The current results revealed low-to-moderate convergence between caregiver and teacher reports on the short-form EFFORT survey. This aligns with previous research on EF behavioral problems (Toplak et al., 2013) and meta-analyses examining parent–teacher rating agreement regarding children’s emotional and behavioral problems (Carneiro et al., 2021; De Los Reyes et al., 2015). Future research should investigate caregiver–teacher agreement across different EF domains, as their salience may vary across home and school settings. Agreement may be higher for behaviors that are more frequently displayed in both settings (e.g., inhibitory control) but lower for behaviors that are more emphasized in the classroom, such as cognitive flexibility.

Further, the assessor reports of sustained attention and behavioral control were related to both caregiver and teacher reports. These associations also ranged from low to moderate, possibly influenced by the setting where assessments were conducted. In Bangladesh, direct assessments were conducted in homes, and assessor ratings demonstrated a higher association with caregiver reports than teacher reports. In contrast, in Argentina and Australia, assessments were conducted in schools, and assessor ratings exhibited a higher association with teacher reports than caregiver reports. It is unclear if differences in convergence emerge in part because children’s behavior during assessment reflects the expectation of the larger setting or because of the assessors’ ability to perceive other relevant behaviors in that setting. These analyses should be replicated across contexts, especially given the hypothesized importance of the assessment context and assessor characteristics on EF task performance (Jukes et al., 2024; Obradović & Willoughby, 2019).

The validity of the EFFORT item bank, especially for longitudinal analyses, is strengthened by the positive associations of all EFFORT composites with age. Since the EFFORT items and associated examples were designed to be age-appropriate for children aged 3–12, most item-level correlations with age are low in magnitude in the full samples. The one exception is the caregiver reports on the item “completes age-appropriate numerical mental calculation.” Associations with age most likely reflect the level of independence with which children engage in specific EF behaviors with older children needing less support than younger children. Given that site-specific samples focused primarily on preschool children, the strength of these associations will likely be greater in studies that include a wider age range of children. Indeed, differences in the magnitudes of age associations between caregiver and teacher reports could be due to the fact that the samples with caregiver reports—Bangladesh, South Africa, and the US sites—included older children.

Around the world, childhood socialization experiences and learning opportunities tend to vary for girls and boys (Best & Puzio, 2019). Gender is often a key predictor of how children’s behaviors and developmentally salient competencies are perceived, valued, and supported, with girls facing many resource inequities in certain cultural contexts (Best & Puzio, 2019; Stromquist, 2007). Since caregiver-, teacher-, and assessor-reported short forms demonstrated full measurement invariance for boys and girls across the full analytic samples, the EFFORT item bank holds promise as a valid tool for investigating gender differences in EF behaviors. Moreover, our study revealed that there were no gender differences in mean levels of the short-form composite for caregiver and assessor reports. Teachers, however, reported a slight advantage for girls in the full analytic sample, which is consistent with a gender bias found for teacher-reported EF behaviors of children in the U.S. (Garcia et al., 2019) and other teacher-reported measures of classroom behaviors (e.g., Bennett et al., 1993; Krkovic et al., 2014). Gender differences across reporters should be further investigated in the site-specific samples to better understand the role of home, school, and assessment settings in differentially socializing, eliciting, or rewarding certain EF behaviors among boys and girls.

Because adult-reported measures capture children’s EF within everyday experiences and activities, behavioral ratings often show only low-to-moderate associations with performance on standardized, decontextualized EF tasks (Toplak et al., 2013). Nevertheless, a degree of convergence supports the idea that these distinct assessment approaches measure a set of related skills and behaviors. The current study demonstrated a level of convergence across all three reporters and various EF tasks in a way that aligns with previous research (e.g., Ahmed et al., 2022; Obradović et al., 2022; Toplak et al., 2013; Willoughby et al., 2019). The variability in these associations may be driven by task-specific characteristics. For transparency, each task block was designed to capture a distinct EF skill and was analyzed separately. This stands in contrast to typical approaches where performance on several different EF tasks is aggregated to achieve a more reliable measure. Furthermore, specific sample characteristics could also contribute to variable convergence. In addition to sample size, the young age of some of the participants from the Global South makes them particularly vulnerable to EF task assumptions (e.g., academic thinking and subject-matter familiarity) that are not culturally universal (Jukes et al., 2024). Finally, we observed a more robust and persistent association of EF tasks with teacher reports than with caregiver reports. This could be driven by the fact that direct assessment of EF skills shares many characteristics with academic testing and performance on structured classroom activities. Associations with assessor ratings were similar to, if not larger than, teacher reports. This further corroborates that the structure of EF assessments, which privileges focused attention, impulse inhibition, and abstract thinking, shares more variance with EF behaviors observed in formal education settings. It is also possible that caregivers, especially those with less formal education, may find it difficult to distinguish nuanced EF behaviors from a child’s general conduct and compliance, which may reflect factors beyond EF skills. These reporter differences warrant further investigation to better understand unique values of different assessment perspectives.

### 4.3. Limitations

The inclusion of samples from seven culturally distinct sites can be viewed as a major strength of our analysis. To make this research feasible, however, it was necessary to include the EFFORT survey in larger, ongoing investigations. This approach introduced inconsistencies across sites in the sample size, sample characteristics (notably, child age), the reporters used for the EFFORT survey, and in the EF direct assessment tasks. For the primary analyses using the full sample, sites with larger sample sizes are weighted more heavily and sites that did not include all reporters are omitted completely from certain analyses (e.g., we could only examine convergence between teacher and caregiver reports at sites that included both). Site differences in results could be partially confounded with child age: in addition to differences in mean age across sites, some sites had a narrow age range relative to others. Expanding the age range in future studies would allow us to investigate whether results differ by age within sites, and to more clearly understand whether EF skills measured by the EFFORT survey differ across sites. In the full sample, the large sample size enabled us to conduct a variety of analyses with a high level of precision and adequate power. Unfortunately, we were not able to repeat these analyses for each site individually due to insufficient sample size at many of the specific sites. Future research would establish measurement invariance for the EFFORT survey within and across cultural contexts.

## 5. Conclusions

The central goal of the current study was to provide a reliable item bank of universal EF behaviors to promote further inquiry into precisely how, when, and for whom these behaviors develop and relate to developmentally salient competences. To promote open science and broader impact, we are making the full bank of caregiver, teacher, and assessor EFFORT items, along with supporting documentation, available under a Creative Commons license so that other researchers are free to use and modify the assessment at no cost (see OSF link https://osf.io/k3edb, accessed on 21 April 2026). The supporting documentation includes all the EFFORT items for caregivers and teachers, item-level translation and adaptation guidelines, and expanded lists of example behaviors. While the EF behavioral factors and composites we created for this paper are a good starting point, we urge researchers to select and adapt items most relevant to their context and inquiry. This process should include the following steps: (1) selection of contextually and study-relevant items; (2) adaptation of culturally specific examples of activities and situations that elicit EF behaviors; (3) translation of selected items according to provided guidelines to ensure that universal aspects of each item are preserved across languages.

Further, we will establish a public OSF data repository to facilitate sharing of de-identified data. This repository will include common demographic data to support the ongoing examination of psychometric properties across different ages, reporters, and cultural contexts. A public data repository will enable cross-context studies of EF behaviors and domains that share cultural relevance to deepen our understanding of how EFs are socialized and promoted in daily activities of young children across diverse cultures.

We hope that this tool will shed light on what aspects of EF behaviors are universal and what are culturally specific. Some scholars have argued that EF skills and behaviors are primarily relevant for children who attend formal schooling (Kroupin et al., 2025). Indeed, most education settings value and promote development and use of EFs, as these behaviors help children pursue learning goals, maintain focused attention, foster peer collaboration, and self-regulate affect according to the classroom rules. Teacher- and assessor-reported EFFORT items can be leveraged to understand the unique value of EF behaviors in supporting various school outcomes. However, the perceived lack of evidence regarding how EF behaviors support goal-directed behaviors beyond the classroom may be a result of limited availability of assessments that center children’s engagement with family and community activities, such as household chores, assistance with errands, or culturally relevant activities (e.g., crafting or dancing, social rituals, religious practices, farming and herding). We hope that caregiver-reported EFFORT items will expand the empirical evidence regarding the importance of EFs in family and community settings that represent diverse cultural experiences and expectations. But we need more assessments, beyond caregiver reports, that capture individual differences in children’s competencies across family, community, and cultural activities. The culturally relevant adaptations of the EFFORT item bank can also be used to validate new EF tasks that center cultural strengths (Gaskins & Alcalá, 2023; Miller-Cotto et al., 2022).

## Figures and Tables

**Table 1:** Item-Level Descriptives for Caregiver and Teacher Reports in the Full Sample.

	Caregiver Items					Teacher Items				
Item No.	*M*	*SD*	Skew	Age Corr	Female Corr	*M*	*SD*	Skew	Age Corr	Female Corr
**A1**	2.99	0.89	−0.41	0.14 ***	0.03	2.89	0.97	−0.35	0.10 ***	0.12 ***
**A2**	2.64	1.01	−0.15	0.17 ***	−0.05	2.85	0.97	−0.34	0.18 ***	0.12 ***
A3	2.79	1.04	−0.28	0.27 ***	−0.01	2.72	1.02	−0.19	0.16 ***	0.12 ***
A4	3.30	0.85	−0.94	0.20 ***	0.06 *	2.86	0.97	−0.34	0.10 ***	0.10 ***
A5	2.91	0.96	−0.39	0.09 ***	0.02	2.64	0.99	−0.15	0.09 ***	0.07 **
**B1**	2.67	1.00	−0.14	0.12 ***	−0.02	2.71	0.93	−0.17	0.12 ***	0.07 *
B2	2.82	1.00	−0.29	0.25 ***	0.03	2.81	0.99	−0.33	0.18 ***	0.12 ***
**B3**	2.87	0.98	−0.36	0.18 ***	−0.02	2.74	1.01	−0.25	0.14 ***	0.11 ***
B4	2.57	1.02	−0.04	0.08 **	0.03	2.66	0.97	−0.14	0.12 ***	0.07 **
B5	2.99	0.95	−0.51	0.20 ***	−0.01	2.56	0.97	−0.07	0.19 ***	0.08 **
C1	2.81	1.01	−0.29	0.15 ***	−0.02	2.60	0.96	−0.06	0.19 ***	0.07 **
**C2**	2.85	0.99	−0.36	0.19 ***	−0.03	2.78	0.95	−0.32	0.19 ***	0.10 ***
C3	2.79	0.99	−0.24	0.14 ***	−0.04	2.78	0.93	−0.25	0.17 ***	0.13 ***
**C4**	2.96	0.98	−0.52	0.27 ***	0.02	2.78	0.97	−0.26	0.24 ***	0.11 ***
C5	3.05	0.97	−0.64	0.10 ***	0.02	2.74	0.99	−0.25	0.05	0.08 **
**D1**	2.89	0.98	−0.40	0.20 ***	0.05	2.85	0.96	−0.38	0.11 ***	0.12 ***
**D2**	2.76	0.99	−0.22	0.22 ***	0.04	2.66	0.98	−0.12	0.15 ***	0.07 **
D3	3.19	0.89	−0.81	0.10 ***	−0.01	2.68	0.99	−0.22	0.11 ***	0.07 **
D4	2.86	1.13	−0.45	0.44 ***	0.02	2.42	1.04	0.06	0.26 ***	0.02
D5	3.00	0.94	−0.52	0.15 ***	0.01	2.66	0.99	−0.14	0.09 ***	0.07 *
D6	3.30	0.87	−1.02	0.18 ***	0.00	2.92	0.97	−0.39	0.07 *	0.05
**E1**	2.97	0.97	−0.45	0.22 ***	0.02	2.45	1.00	0.03	0.18 ***	0.04
E2	2.89	1.02	−0.37	0.24 ***	−0.01	2.48	0.98	0.00	0.18 ***	0.07 **
**E3**	2.84	0.99	−0.36	0.17 ***	−0.01	2.74	0.98	−0.19	0.13 ***	0.04
E4	2.77	0.97	−0.24	0.15 ***	−0.01	2.60	0.97	0.00	0.04	0.06 *
E5	2.90	1.00	−0.42	0.13 ***	0.06 *	2.67	1.01	−0.19	0.16 ***	0.09 **
E6	2.98	0.94	−0.45	0.22 ***	0.03	2.69	0.93	−0.16	0.17 ***	0.13 ***
F1	3.16	0.92	−0.80	0.12 ***	0.02	2.87	0.96	−0.42	0.03	0.12 ***
F2	2.65	1.03	−0.15	0.21 ***	0.01	2.50	1.01	0.05	0.16 ***	0.09 ***
**F3**	3.03	0.96	−0.58	0.11 ***	−0.02	2.86	1.02	−0.42	0.14 ***	0.06 *
F4	2.85	1.06	−0.42	0.07 *	0.01	2.70	1.04	−0.22	0.13 ***	0.06 *
**F5**	2.70	1.00	−0.25	0.04	0.04	2.78	1.00	−0.26	0.10 ***	0.08 **

Note. * *p* < 0.05; ** *p* < 0.01; *** *p* < 0.001. *N* (teacher) = 892; *N* (caregiver) = 1286. Bold items in short form.

**Table 2:** Item-Level Descriptives for Assessor Reports in the Full Sample.

Assessor Survey					
Item	M	SD	Skew	Age Corr	Female Corr
1	3.06	0.98	−0.66	0.23 ***	0.02
2	3.03	1.00	−0.63	0.23 ***	0.01
3	2.99	1.02	−0.57	0.18 ***	0.00
4	2.95	1.02	−0.49	0.21 ***	0.03
5	2.89	0.97	−0.38	0.16 ***	0.00
6	3.18	0.93	−0.85	0.19 ***	0.02
7	3.36	0.86	−1.21	0.13 ***	0.03
8	3.36	0.90	−1.22	0.17 ***	0.01
9	3.27	0.89	−1.02	0.17 ***	0.03
10	2.58	1.08	−0.09	0.11 ***	0.02

Note. *** *p* < 0.001. *N* = 1291.

**Table 3:** Six-Factor CFA Model Results for Caregiver and Teacher 32-Item Reports.

	Full Sample		Australia		Bangladesh	Haiti	Sri Lanka		U.S.
	C	T	C	T	C	T	C	T	C
*N*	1286	892	218	227	448	353	155	166	293
Age, *M* (*SD*)	6.73 (2.20)	5.57 (1.25)	4.68 (0.51)	4.69 (0.52)	7.97 (1.99)	5.29 (0.71)	6.61 (0.59)	6.61 (0.60)	7.85 (2.02)
% female	48.0	47.6	42.5	44.1	50.0	50.6	44.5	44.1	47.8
Chi Sq	1519.77	1441.76	714.98	1045.73	754.25	800.38	781.21	954.87	815.87
df	450	449	446	448	448	449	449	449	449
CFI	0.923	0.931	0.905	0.903	0.916	0.934	0.725	0.929	0.925
RMSEA	0.043	0.050	0.053	0.077	0.039	0.047	0.069	0.095	0.053
**Item**	**Factor 1: Attentional Focus/Engagement**								
A1	0.65	0.81	0.73	0.86	0.57	0.81	0.50	0.85	0.73
A2	0.54	0.79	0.46	0.77	0.48	0.82	0.62	0.86	0.46
A3	0.68	0.80	0.50	0.75	0.58	0.84	0.51	0.86	0.51
A4	0.62	0.79	0.76	0.85	0.46	0.77	0.31	0.81	0.76
A5	0.66	0.74	0.56	0.70	0.66	0.78	0.49	0.81	0.55
*Alpha*	0.79	0.89	0.80	0.90	0.69	0.90	0.62	0.93	0.84
**Item**	**Factor 2: Inhibitory Control—Interference Suppression**								
B1	0.60	0.67	0.70	0.83	0.59	0.49	0.44	0.83	0.74
B2	0.71	0.78	0.72	0.84	0.56	0.76	0.60	0.87	0.75
B3	0.70	0.78	0.55	0.71	0.61	0.76	0.60	0.87	0.55
B4	0.60	0.65	0.83	0.89	0.49	0.50	0.51	0.83	0.83
B5	0.64	0.71	0.52	0.63	0.58	0.71	0.61	0.85	0.51
*Alpha*	0.78	0.84	0.80	0.88	0.69	0.78	0.69	0.93	0.83
**Item**	**Factor 3: Inhibitory Control—Response Inhibition**								
C1	0.63	0.74	0.66	0.76	0.62	0.69	0.50	0.87	0.64
C2	0.71	0.78	0.77	0.85	0.58	0.73	0.50	0.78	0.77
C3	0.68	0.77	0.68	0.84	0.55	0.76	0.50	0.81	0.68
C4	0.70	0.76	0.58	0.73	0.62	0.76	0.51	0.82	0.60
C5	0.67	0.71	0.71	0.70	0.64	0.69	0.60	0.84	0.70
*Alpha*	0.81	0.86	0.81	0.88	0.75	0.84	0.64	0.91	0.86
**Item**	**Factor 4: Working Memory**								
D1	0.64	0.78	0.52	0.73	0.62	0.73	0.51	0.88	0.52
D2	0.71	0.82	0.72	0.82	0.63	0.79	0.62	0.89	0.72
D3	0.65	0.79	0.62	0.83	0.65	0.78	0.45	0.86	0.62
D4	0.60	0.70	0.51	0.67	0.65	0.68	0.55	0.88	0.51
D5	0.65	0.74	0.64	0.76	0.50	0.75	0.53	0.82	0.64
D6	0.62	0.61	0.53	0.70	0.51	0.56	0.50	0.85	0.52
*Alpha*	0.80	0.88	0.76	0.89	0.76	0.86	0.69	0.94	0.86
**Item**	**Factor 5: Cognitive Flexibility**								
E1	0.65	0.76	0.66	0.83	0.49	0.61	0.35	0.91	0.66
E2	0.67	0.72	0.58	0.78	0.59	0.68	0.62	0.91	0.58
E3	0.61	0.70	0.59	0.84	0.44	0.63	0.58	0.87	0.59
E4	0.66	0.72	0.77	0.79	0.69	0.66	0.51	0.88	0.77
E5	0.52	0.74	0.69	0.81	0.45	0.67	0.42	0.86	0.69
E6	0.72	0.75	0.78	0.80	0.57	0.69	0.60	0.83	0.78
*Alpha*	0.80	0.87	0.84	0.92	0.70	0.82	0.69	0.95	0.87
**Item**	**Factor 6: Planning & Organization**								
F1	0.68	0.73	0.68	0.83	0.58	0.69	0.58	0.89	0.68
F2	0.65	0.74	0.57	0.83	0.52	0.63	0.58	0.91	0.57
F3	0.72	0.71	0.56	0.65	0.71	0.70	0.58	0.85	0.56
F4	0.65	0.72	0.53	0.75	0.68	0.70	0.60	0.89	0.53
F5	0.60	0.72	0.68	0.81	0.53	0.64	0.54	0.80	0.68
*Alpha*	0.82	0.86	0.78	0.89	0.76	0.83	0.72	0.94	0.87

Note. C = caregiver; T = teacher. One factor correlation (between response inhibition and working memory) for caregivers was estimated to be slightly greater than 1.00 and was consequently fixed to 1.0. In the Bangladesh caregiver model, three residual covariances were added (B4~~C3, A2~~D1, A2~~F5). In the Australia caregiver model, three residual covariances were added (B1~~B2, B3~~C4, A3~~D5).

**Table 4:** Two-factor CFA Model Results for Assessor Reports.

	Full Sample	Australia	Bangladesh	Haiti	South Africa
*N*	1211	261	448	338	100
Age, *M* (*SD*)	6.50 (2.20)	4.68 (0.51)	7.97 (1.99)	5.29 (0.72)	8.26 (3.46)
Chi Sq	239.38	103.40	157.18	81.29	7.96
df	34	34	34	34	34
CFI	0.968	0.957	0.959	0.957	0.890
RMSEA	0.067	0.088	0.090	0.064	0.107
SRMR	0.026	0.043	0.028	0.036	0.061
**Item No.**	**Factor 1: Sustained Attention**				
1. Pays attention	0.87	0.85	0.90	0.79	0.81
2. Finishes tasks	0.89	0.89	0.90	0.84	0.80
3. Remains focused	0.90	0.93	0.95	0.83	0.78
4. Persists	0.87	0.89	0.89	0.83	0.72
5. Ignores distraction	0.79	0.68	0.87	0.80	0.60
10. Checks mistakes	0.72	0.65	0.78	0.80	0.48
*Alpha*	0.94	0.92	0.95	0.92	0.85
**Item No.**	**Factor 2: Behavioral Control**				
6. Doesn’t rush	0.87	0.82	0.88	0.86	0.70
7. Waits to speak	0.77	0.61	0.87	0.88	0.65
8. Stops behavior	0.77	0.67	0.79	0.86	0.59
9. Patiently awaits	0.88	0.85	0.87	0.89	0.81
*Alpha*	0.89	0.82	0.91	0.92	0.78
Factor Correlation	0.83 ***	0.76 ***	0.89 ***	0.87 ***	0.80 ***

Note. *** *p* < 0.001.

**Table 5:** One-Factor CFA Model Results for Caregiver and Teacher 12-Item Reports.

	Full Sample		Australia		Bangladesh	Haiti	Sri Lanka		U.S.
Report	C	T	C	T	C	T	C	T	C
*N*	1264	892	218	227	448	353	155	166	293
Chi Sq	234.28	171.91	104.87	186.21	98.92	126.94	72.63	282.00	97.18
df	54	54	53	54	54	54	54	54	54
CFI	0.952	0.973	0.932	0.930	0.954	0.949	0.923	0.946	0.969
RMSEA	0.051	0.049	0.067	0.104	0.043	0.062	0.049	0.105	0.053
SRMR	0.034	0.026	0.052	0.042	0.038	0.044	0.061	0.033	0.035
**Item No.**	**Factor Loadings and Cronbach’s Alpha**								
A1	0.66	0.77	0.72	0.84	0.61	0.76	0.49	0.85	0.74
A2	0.58	0.78	0.44	0.78	0.58	0.81	0.70	0.88	0.73
B1	0.62	0.66	0.70	0.77	0.60	0.49	0.51	0.83	0.58
B3	0.69	0.78	0.56	0.77	0.62	0.72	0.60	0.87	0.78
C2	0.64	0.69	0.77	0.86	0.48	0.56	0.48	0.75	0.60
C4	0.64	0.71	0.56	0.79	0.56	0.60	0.37	0.82	0.69
D1	0.66	0.78	0.52	0.73	0.65	0.74	0.47	0.89	0.80
D2	0.68	0.80	0.74	0.82	0.59	0.77	0.50	0.87	0.76
E1	0.56	0.72	0.62	0.82	0.41	0.57	0.31	0.89	0.55
E3	0.58	0.67	0.51	0.79	0.43	0.59	0.59	0.85	0.65
F3	0.62	0.68	0.43	0.57	0.63	0.71	0.38	0.86	0.79
F5	0.55	0.70	0.53	0.80	0.54	0.61	0.48	0.80	0.68
*Alpha*	0.88	0.93	0.87	0.95	0.84	0.90	0.78	0.97	0.92
Correlation with Full Form	0.96 ***	0.98 ***	0.99 ***	1.00 ***	0.95 ***	0.97 ***	0.99 ***	0.99 ***	0.97 ***

Note. One residual covariance added in Australia caregiver model: B3~~C4. One residual covariance added in the Sri Lanka caregiver model: D1~~F3. The Sri Lanka teacher model was fit without SE clustering at the teacher level due to model convergence issues. *** *p* < 0.001.

**Table 6:** Gender Invariance for 12-item short form.

	Teacher Report			Caregiver Report			Assessor Report		
	Configural	Metric	Scalar	Configural	Metric	Scalar	Configural	Metric	Scalar
χ^2^	218.77	230.05	246.28	301.81	321.99	348.31	273.19	288.50	302.25
DF	108	119	130	108	119	130	68	76	84
Satorra–Bentler χ^2^ Difference	NA	4.96	20.01	NA	14.04	39.79	NA	4.25	4.89
Comp. *p*-value	NA	0.93	0.64	NA	0.21	0.023	NA	0.83	0.77
CFI	0.972	0.972	0.971	0.952	0.949	0.946	0.967	0.966	0.965
ΔCFI	NA	<0.001	0.001	NA	0.003	0.003	NA	0.001	0.001
RMSEA	0.050	0.048	0.047	0.052	0.051	0.050	0.068	0.066	0.063
SRMR	0.030	0.034	0.035	0.037	0.043	0.044	0.028	0.031	0.031

Note. NA = not applicable.

**Table 7:** EFFORT Correlations Among Reporters.

		Teacher		Caregiver	
Site	Reporter	*r*	*n*	*r*	*n*
Full sample	Caregiver	0.27 ***	419		
	Assessor Attention	0.23 ***	727	0.50 ***	876
	Assessor Behavior	0.25 ***	727	0.51 ***	876
Argentina	Caregiver	0.38 *	49		
	Assessor Attention	0.57 ***	41	0.26 ^†^	46
	Assessor Behavior	0.56 ***	41	0.27 ^†^	46
Australia	Caregiver	0.38 ***	217		
	Assessor Attention	0.31 ***	217	0.14 *	206
	Assessor Behavior	0.35 ***	217	0.15 *	206
Bangladesh	Caregiver	0.42 ***	58		
	Assessor Attention	0.26 *	58	0.48 ***	448
	Assessor Behavior	0.28 *	58	0.50 **	448
Haiti	Assessor Attention	0.24 ***	338	NA	
	Assessor Behavior	0.21 ***	338	NA	
South Africa	Assessor Attention	NA		0.53 ***	100
	Assessor Behavior	NA		0.43 ***	100
Sri Lanka	Caregiver	0.26 **	140		

Note. ^†^ *p* < 0.1, * *p* < 0.05, ** *p* < 0.01, *** *p* < 0.001.; NA = not applicable.

**Table 8:** EF Direct Assessment Measures Regressed on EFFORT Short-Form Composites.

Site/Task	Construct			
Argentina	Caregiver	Teacher	Assessor Attention	Assessor Behavior
H&F—flower trials	0.24 (*N* = 38)	0.39 * (*N* = 36)	0.64 *** (*N* = 37)	0.61 *** (*N* = 37)
H&F—mixed trials accuracy	0.11 (*N* = 39)	0.43 ** (*N* = 36)	0.68 *** (*N* = 38)	0.71 *** (*N* = 38)
Memory Game forward correct	0.08 (*N* = 39)	0.33 * (*N* = 35)	0.19 (*N* = 39)	0.19 (*N* = 39)
Memory Game backwards correct	0.06 (*N* = 39)	0.33 ^†^ (*N* = 35)	0.31 ^†^ (*N* = 39)	0.31 ^†^ (*N* = 39)
**Australia**	**Caregiver**	**Teacher**	**Assessor** **Attention**	**Assessor** **Behavior**
Go/No-Go	0.09 (*N* = 208)	0.19 ** (*N* = 199)	0.48 *** (*N* = 234)	0.35 *** (*N* = 234)
Head–Toes–Knees–Shoulders	0.21 ** (*N* = 213)	0.26 *** (*N* = 203)	0.41 *** (*N* = 238)	0.28 *** (*N* = 238)
**Bangladesh**	**Caregiver**	**Teacher**	**Assessor** **Attention**	**Assessor** **Behavior**
H&F—flower trials	0.15 *** (*N* = 419)	0.14 (*N* = 54)	0.29 *** (*N* = 419)	0.26 *** (*N* = 419)
H&F—mixed trials accuracy	0.17 *** (*N* = 416)	0.08 (*N* = 53)	0.38 *** (*N* = 416)	0.29 *** (*N* = 416)
Memory Game forward correct	0.15 ** (*N* = 421)	0.28 * (*N* = 54)	0.40 *** (*N* = 421)	0.32 *** (*N* = 421)
Memory Game backwards correct	0.06 (*N* = 420)	0.26 * (*N* = 54)	0.35 *** (*N* = 420)	0.27 *** (*N* = 420)
**Haiti**	**Caregiver**	**Teacher**	**Assessor** **Attention**	**Assessor** **Behavior**
Head–Toes–Knees–Shoulders (IDELA)	---	0.21 *** (*N* = 327)	0.21 *** (*N* = 327)	0.10 ^†^ (*N* = 327)
Forward memory span (IDELA)	---	0.08 (*N* = 327)	0.10 ^†^ (*N* = 327)	0.12 * (*N* = 327)
**South Africa**	**Caregiver**	**Teacher**	**Assessor** **Attention**	**Assessor** **Behavior**
Forward memory span	0.17 * (*N* = 86)	---	0.31 *** (*N* = 82)	0.11 (*N* = 82)
Backward memory span	0.28 * (*N* = 68)	---	0.15 (*N* = 64)	0.23 ^†^ (*N* = 64)
H&F—flower trials	0.12 (*N* = 97)	---	0.28 ** (*N* = 97)	0.34 ** (*N* = 94)
H&F—mixed trials accuracy	0.26 * (*N* = 97)	---	0.37 *** (*N* = 97)	0.28 *** (*N* = 94)

Note. Beta coefficients are standardized. All models control for gender and age. ^†^ *p* < 0.1, * *p* < 0.05, ** *p* < 0.01, *** *p* < 0.001. Participants who had accuracy lower than 50% in the hearts trials were excluded from analysis.

## Data Availability

The datasets presented in this article are not readily available because the data are part of ongoing longitudinal research at some of the sites. Requests to access the datasets should be directed to Jelena Obradović (obradovic-lab@stanford.edu).

## Supplementary Materials

The following supporting information can be downloaded at: https://www.mdpi.com/article/10.3390/bs16050693/s1, Figure S1: Teacher Item Histograms; Figure S2: Caregiver Item Histograms; Figure S3: Assessor Item Histograms; Figure S4: Image used to facilitate family responses during EFFORT administration; Table S1: EFFORT Teacher and Caregiver Report; Table S2: EFFORT Assessor Report; Table S3: Number of Observations for Study Samples; Table S4: Site Level Study Characteristics; Text S1: Translation and Adaptation of EFFORT.

## References

[B1-behavsci-16-00693] Achenbach T. M. (1991). Manual for child behavior checklist/4-18 and 1991 profile.

[B2-behavsci-16-00693] Ahmed I., Obradović J. (2026). A movement-based working memory measure rooted in observational learning: Relevance for cognitive skills and everyday behaviors.

[B3-behavsci-16-00693] Ahmed I., Steyer L., Suntheimer N. M., Wolf S., Obradović J. (2022). Directly assessed and adult-reported executive functions: Associations with academic skills in Ghana. Journal of Applied Developmental Psychology.

[B4-behavsci-16-00693] Amukune S., Józsa K. (2021). The childhood executive functioning inventory (CHEXI): Psychometric properties and association with academic achievement in Kenyan first graders. Journal of Psychological and Educational Research.

[B5-behavsci-16-00693] Bennett R. E., Gottesman R. L., Rock D. A., Cerullo F. (1993). Influence of behavior perceptions and gender on teachers’ judgments of students’ academic skill. Journal of Educational Psychology.

[B6-behavsci-16-00693] Best D. L., Puzio A. R. (2019). Gender and culture. The handbook of culture and psychology.

[B7-behavsci-16-00693] Betancourt T. S., Speelman L., Onyango G., Bolton P. (2009). A qualitative study of mental health problems among children displaced by war in northern Uganda. Transcultural Psychiatry.

[B8-behavsci-16-00693] Blumenthal A., Ghalibaf E., Pitiot A., Hirst R., Cook J., Balume B., Akilamali J. P., Makaula H., Sikweyiya N., Draper C., Blanchette I. (2026). Zola bongo: Enabling offline tablet-based cognitive testing in diverse populations.

[B9-behavsci-16-00693] Bolz W. (2002). Psychological analysis of the Sri Lankan conflict culture with special reference to the high suicide rate. Crisis.

[B10-behavsci-16-00693] Bornstein M. H., Sameroff A. (2009). Toward a model of culture↔parent↔child transactions. The transactional model of development: How children and contexts shape each other.

[B11-behavsci-16-00693] Brandmiller C., Dumont H., Becker M. (2020). Teacher perceptions of learning motivation and classroom behavior: The role of student characteristics. Contemporary Educational Psychology.

[B12-behavsci-16-00693] Camerota M., Willoughby M. T., Kuhn L. J., Blair C. B. (2018). The childhood executive functioning inventory (CHEXI): Factor structure, measurement invariance, and correlates in US preschoolers. Child Neuropsychology.

[B13-behavsci-16-00693] Carneiro A., Soares I., Rescorla L., Dias P. (2021). Meta-analysis on parent-teacher agreement on preschoolers’ emotional and behavioural problems. Child Psychiatry and Human Development.

[B14-behavsci-16-00693] Chen F. F. (2007). Sensitivity of goodness of fit indexes to lack of measurement invariance. Structural Equation Modeling: A Multidisciplinary Journal.

[B15-behavsci-16-00693] Cortés Pascual A., Moyano Muñoz N., Quílez Robres A. (2019). The relationship between executive functions and academic performance in primary education: Review and meta-analysis. Frontiers in Psychology.

[B16-behavsci-16-00693] Croucher S. M., Bruno A., McGrath P., Adams C., McGahan C., Suits A., Huckins A. (2012). Conflict styles and high–low context cultures: A cross-cultural extension. Communication Research Reports.

[B17-behavsci-16-00693] Davidson M. C., Amso D., Anderson L. C., Diamond A. (2006). Development of cognitive control and executive functions from 4 to 13 years: Evidence from manipulations of memory, inhibition, and task switching. Neuropsychologia.

[B18-behavsci-16-00693] Dekker M. C., Ziermans T. B., Spruijt A. M., Swaab H. (2017). Cognitive, parent and teacher rating measures of executive functioning: Shared and unique influences on school achievement. Frontiers in Psychology.

[B19-behavsci-16-00693] De Los Reyes A., Augenstein T. M., Wang M., Thomas S. A., Drabick D. A., Burgers D. E., Rabinowitz J. (2015). The validity of the multi-informant approach to assessing child and adolescent mental health. Psychological Bulletin.

[B20-behavsci-16-00693] Diamond A. (2013). Executive functions. Annual Review of Psychology.

[B21-behavsci-16-00693] Doyle A. E., Willcutt E. G., Seidman L. J., Biederman J., Chouinard V.-A., Silva J., Faraone S. V. (2005). Attention-Deficit/hyperactivity disorder endophenotypes. Biological Psychiatry.

[B22-behavsci-16-00693] Duncan G. J., Dowsett C. J., Claessens A., Magnuson K., Huston A. C., Klebanov P., Pagani L. S., Feinstein L., Engel M., Brooks-Gunn J., Sexton H., Duckworth K., Japel C. (2007). School readiness and later achievement. Developmental Psychology.

[B23-behavsci-16-00693] Eisenberg N., Spinrad T. L., Fabes R. A., Reiser M., Cumberland A., Shepard S. A., Valiente C., Losoya S. H., Guthrie I. K., Thompson M. (2004). The relations of effortful control and impulsivity to children’s resiliency and adjustment. Child Development.

[B24-behavsci-16-00693] Ezeugwu C., Oyekola A., Ayede A. (2025). Members of the Nigeria Child Development Research Network Broadening cognitive science in Nigeria: Foundation for a new discipline. Cognitive Science.

[B25-behavsci-16-00693] Fay-Stammbach T., Hawes D. J., Meredith P. (2014). Parenting influences on executive function in early childhood: A review. Child Development Perspectives.

[B26-behavsci-16-00693] Finders J. K., McClelland M. M., Geldhof G. J., Rothwell D. W., Hatfield B. E. (2021). Explaining achievement gaps in kindergarten and third grade: The role of self-regulation and executive function skills. Early Childhood Research Quarterly.

[B27-behavsci-16-00693] Fitzpatrick C., Côté-Lussier C., Blair C. (2016). Dressed and groomed for success in elementary school: Student appearance and academic adjustment. The Elementary School Journal.

[B28-behavsci-16-00693] Garcia E. B., Sulik M. J., Obradović J. (2019). Teachers’ perceptions of students’ executive functions: Disparities by gender, ethnicity, and ELL status. Journal of Educational Psychology.

[B29-behavsci-16-00693] Garon N., Bryson S. E., Smith I. M. (2008). Executive function in preschoolers: A review using an integrative framework. Psychological Bulletin.

[B30-behavsci-16-00693] Gaskins S., Alcalá L. (2023). Studying executive function in culturally meaningful ways. Journal of Cognition and Development.

[B31-behavsci-16-00693] Gioia G. A., Isquith P. K., Guy S. C., Kenworthy L. (2000). Behavior rating inventory of executive function. Child Neuropsychology.

[B32-behavsci-16-00693] Gioia G. A., Isquith P. K., Kenworthy L., Barton R. M. (2002). Profiles of everyday executive function in acquired and developmental disorders. Child Neuropsychology.

[B33-behavsci-16-00693] Hermida M. J., Lipina S. J., Segretin M. S. (2025). Temperament ratings by parents and teachers as predictors of non-verbal ability in argentinean preschoolers. Infant and Child Development.

[B34-behavsci-16-00693] Howard S. J., Melhuish E. (2017). An early years toolbox for assessing early executive function, language, self-regulation, and social development: Validity, reliability, and preliminary norms. Journal of Psychoeducational Assessment.

[B35-behavsci-16-00693] Hu L., Bentler P. M. (1998). Fit indices in covariance structure modeling: Sensitivity to underparameterized model misspecification. Psychological Methods.

[B36-behavsci-16-00693] Hu L., Bentler P. M. (1999). Cutoff criteria for fit indexes in covariance structure analysis: Conventional criteria versus new alternatives. Structural Equation Modeling: A Multidisciplinary Journal.

[B37-behavsci-16-00693] Hughes C. (2023). Executive functions: Going places at pace. Journal of Cognition and Development.

[B38-behavsci-16-00693] Joyner K. B., Silver C. H., Stavinoha P. L. (2009). Relationship between parenting stress and ratings of executive functioning in children with ADHD. Journal of Psychoeducational Assessment.

[B39-behavsci-16-00693] Jukes M. C. H., Ahmed I., Baker S., Draper C. E., Howard S. J., McCoy D. C., Obradović J., Wolf S. (2024). Principles for adapting assessments of executive function across cultural contexts. Brain Sciences.

[B40-behavsci-16-00693] Kraemer H. C., Measelle J. R., Ablow J. C., Essex M. J., Boyce W. T., Kupfer D. J. (2003). A new approach to integrating data from multiple informants in psychiatric assessment and research: Mixing and matching contexts and perspectives. American Journal of Psychiatry.

[B41-behavsci-16-00693] Krkovic K., Greiff S., Kupiainen S., Vainikainen M.-P., Hautamäki J. (2014). Teacher evaluation of student ability: What roles do teacher gender, student gender, and their interaction play?. Educational Research.

[B42-behavsci-16-00693] Kroupin I., Davis H. E., Burdett E., Cuata A. B., Hartley V., Henrich J. (2025). The cultural construction of “executive function”. Proceedings of the National Academy of Sciences of the United States of America.

[B43-behavsci-16-00693] Lansford J. E. (2022). Annual research review: Cross-cultural similarities and differences in parenting. Journal of Child Psychology and Psychiatry.

[B44-behavsci-16-00693] Lightbody A., Sparks B., Frank M. (2026). Development and initial validation of the LEVANTE caregiver report surveys.

[B45-behavsci-16-00693] Mahone E. M., Hagelthorn K. M., Cutting L. E., Schuerholz L. J., Pelletier S. F., Rawlins C., Singer H. S., Denckla M. B. (2002). Effects of IQ on executive function measures in children with ADHD. Child Neuropsychology.

[B46-behavsci-16-00693] McClelland M. M., Cameron C. E. (2012). Self-regulation in early childhood: Improving conceptual clarity and developing ecologically valid measures. Child Development Perspectives.

[B47-behavsci-16-00693] McClelland M. M., Cameron C. E., Duncan R., Bowles R. P., Acock A. C., Miao A., Pratt M. E. (2014). Predictors of early growth in academic achievement: The head-toes-knees-shoulders task. Frontiers in Psychology.

[B48-behavsci-16-00693] McCoy D. C. (2019). Measuring young children’s executive function and self-regulation in classrooms and other real-world settings. Clinical Child and Family Psychology Review.

[B49-behavsci-16-00693] McCoy D. C., Sabol T. J. (2025). Overcoming the streetlight effect: Shining light on the foundations of learning and development in early childhood. American Psychologist.

[B50-behavsci-16-00693] McHenry M. S., Mukherjee D., Bhavnani S., Kirolos A., Piper J. D., Crespo-Llado M. M., Gladstone M. J. (2023). The current landscape and future of tablet-based cognitive assessments for children in low-resourced settings. PLoS Digital Health.

[B51-behavsci-16-00693] Messer E. J. E., Roome H. E., Legare C. H. (2025). Learning to control through culture: Explaining variation in the development of self-regulation. Psychological Review.

[B52-behavsci-16-00693] Miller-Cotto D., Smith L. V., Wang A. H., Ribner A. D. (2022). Changing the conversation: A culturally responsive perspective on executive functions, minoritized children and their families. Infant and Child Development.

[B53-behavsci-16-00693] Miyake A., Friedman N. P., Emerson M. J., Witzki A. H., Howerter A., Wager T. D. (2000). The unity and diversity of executive functions and their contributions to complex “frontal lobe” tasks: A latent variable analysis. Cognitive Psychology.

[B54-behavsci-16-00693] Nilsen E. S., Huyder V., McAuley T., Liebermann D. (2017). Ratings of everyday executive functioning (REEF): A parent-report measure of preschoolers’ executive functioning skills. Psychological Assessment.

[B55-behavsci-16-00693] Obradović J. (2019). Assessment of motivation, effort, and self-regulation (AMES).

[B56-behavsci-16-00693] Obradović J., Finch J. E., Connolly C., Siyal S., Yousafzai A. K. (2022). The unique relevance of executive functions and self-regulation behaviors for understanding early childhood experiences and preschoolers’ outcomes in rural Pakistan. Developmental Science.

[B57-behavsci-16-00693] Obradović J., Willoughby M. T. (2019). Studying executive function skills in young children in low- and middle-income countries: Progress and directions. Child Development Perspectives.

[B58-behavsci-16-00693] Palmer A. R., Kalstabakken A. W., Distefano R., Carlson S. M., Putnam S. P., Masten A. S. (2025). A short executive functioning questionnaire in the context of early childhood screening: Psychometric properties. Child Neuropsychology: A Journal on Normal and Abnormal Development in Childhood and Adolescence.

[B59-behavsci-16-00693] Ready D. D., Wright D. L. (2011). Accuracy and inaccuracy in teachers’ perceptions of young children’s cognitive abilities: The role of child background and classroom context. American Educational Research Journal.

[B60-behavsci-16-00693] Rothbart M. K., Ahadi S. A., Hershey K., Fisher P. (2001). Investigations of temperament at three to seven years: The children’s behavior questionnaire. Child Development.

[B61-behavsci-16-00693] Silver C. H. (2014). Sources of data about children’s executive functioning: Review and commentary. Child Neuropsychology.

[B62-behavsci-16-00693] Smith-Donald R., Raver C. C., Hayes T., Richardson B. (2007). Preliminary construct and concurrent validity of the preschool self-regulation assessment (PSRA) for field-based research. Early Childhood Research Quarterly.

[B63-behavsci-16-00693] Stromquist N. (2007). The gender socialization process in schools: A cross-national comparison. Paper commissioned for the EFA global monitoring report 2008.

[B64-behavsci-16-00693] Sulik M. J., Obradović J. (2017). Executive functions and externalizing symptoms: Common and unique associations. Journal of Abnormal Child Psychology.

[B65-behavsci-16-00693] Sulik M. J., Obradović J. (2018). Teachers’ rankings of children’s executive functions: Validating a methodology for school-based data collection. Journal of Experimental Child Psychology.

[B66-behavsci-16-00693] Teglasi H., Schussler L., Gifford K., Annotti L. A., Sanders C., Liu H. (2015). Child behavior questionnaire–short form for teachers: Informant correspondences and divergences. Assessment.

[B67-behavsci-16-00693] Thorell L. B., Nyberg L. (2008). The childhood executive functioning inventory (CHEXI): A new rating instrument for parents and teachers. Developmental Neuropsychology.

[B68-behavsci-16-00693] Thorell L. B., Veleiro A., Siu A. F. Y., Mohammadi H. (2013). Examining the relation between ratings of executive functioning and academic achievement: Findings from a cross-cultural study. Child Neuropsychology.

[B69-behavsci-16-00693] Thulin E. J., McLean K. E., Sevalie S., Akinsulure-Smith A. M., Betancourt T. S. (2022). Mental health problems among children in Sierra Leone: Assessing cultural concepts of distress. Transcultural Psychiatry.

[B70-behavsci-16-00693] Toplak M. E., West R. F., Stanovich K. E. (2013). Practitioner review: Do performance-based measures and ratings of executive function assess the same construct?. Journal of Child Psychology and Psychiatry.

[B71-behavsci-16-00693] Ursache A., Blair C., Raver C. C. (2012). The promotion of self-regulation as a means of enhancing school readiness and early achievement in children at risk for school failure. Child Development Perspectives.

[B72-behavsci-16-00693] Wei W. S., McCoy D. C., Busby A. K., Hanno E. C., Sabol T. J. (2021). Beyond neighborhood socioeconomic status: Exploring the role of neighborhood resources for preschool classroom quality and early childhood development. American Journal of Community Psychology.

[B73-behavsci-16-00693] Wiebe S. A., Espy K. A., Charak D. (2008). Using confirmatory factor analysis to understand executive control in preschool children: I. latent structure. Developmental Psychology.

[B74-behavsci-16-00693] Willoughby M. T., Piper B., Oyanga A., Merseth King K. (2019). Measuring executive function skills in young children in Kenya: Associations with school readiness. Developmental Science.

[B75-behavsci-16-00693] Wolf E. J., Harrington K. M., Clark S. L., Miller M. W. (2013). Sample size requirements for structural equation models: An evaluation of power, bias, and solution propriety. Educational and Psychological Measurement.

[B76-behavsci-16-00693] Wolf S., McCoy D. C. (2019). The role of executive function and social-emotional skills in the development of literacy and numeracy during preschool: A cross-lagged longitudinal study. Developmental Science.

[B77-behavsci-16-00693] Yoder M. L., Williford A. P. (2019). Teacher perception of preschool disruptive behavior: Prevalence and contributing factors. Early Education and Development.

[B78-behavsci-16-00693] Zelazo P. D. (2020). Executive function and psychopathology: A neurodevelopmental perspective. Annual Review of Clinical Psychology.

