# Supplementary material for: Executive Function from Observation and Reflection Tool (EFFORT): Validation of a Culturally Adaptable and Publicly Available Item Bank in Seven Countries
Source: Behav Sci (Basel). 2026 Apr 30;16(5):693. doi: 10.3390/bs16050693 (PMC13203204; doi:10.3390/bs16050693)
Supplement: Supplementary file 1 [file behavsci-16-00693-s001.zip › behavsci-4191049-supplementary.pdf]

Table S1

EFFORT Teacher and Caregiver Report

Bold Item Numbers indicate items included in short form analyses.

Instructions: We are interested how often, independently, and easily the student shows each behavior when occasions call for it. Children who excel at a specific behavior should receive 4 (the highest rating), whereas most children will be between 3 and 2. Those who are still developing and need a lot of support should receive 1.

Response Scale:

4 = Always on their own (No support needed)
3 = Mostly on their own (Brief reminders or support)
2 = Sometimes on their own (Regular reminders or moderate support needed)
1 = Not able on their own (Requires a lot of support)

A. Attentional Focus, Engagement, & Persistence

**Item No.**	**Teacher Report**	**Parent Report**
**A1**	**Pays attention when a teacher is explaining or showing something** ... (by listening carefully and/or watching with appropriate eye gaze/body orientation.)	**Pays attention when an adult is explaining or showing something** ... (by listening carefully and/or watching with appropriate eye gaze/body orientation.)
**A2**	**Finishes an assigned task that is repetitive** (e.g., practicing handwriting, cleaning up classroom)	**Finishes an assigned task that is repetitive** (e.g., picking up toys, putting away laundry)
A3	**Remains focused and engaged during lengthy teacher-led activities** (e.g., lecture or story time) by listening carefully and contributing when appropriate.	**Remains focused and engaged during lengthy adult-led activities** (e.g., religious or performance event, family meal time) by listening carefully and contributing when appropriate.
A4	**Remains focused and engaged during collaborative child-led activities** (e.g., art project, peer reading)	**Remains focused and engaged during collaborative child-led activities** (e.g., playing with siblings/peers)
A5	**Persists on a difficult academic/learning task** by trying to figure things out, asking questions, and not quitting	**Persists on a difficult cognitive/learning** task (e.g., homework) by trying to figure things out, asking questions, and not quitting

B. Inhibitory Control - Interference Suppression (aka Ignoring Distractions)

Item No.	Teacher Report	Parent Report
**B1**	**Ignores irrelevant activity/noise** (e.g., traffic, play in the school yard, classroom conversation) **when working on an assigned task**	**Ignores irrelevant activity/noise** (e.g., crying sibling, TV, outside traffic) **when working on an assigned task**
B2	**Completes an assigned task before moving to a next task**	**Completes an assigned task before moving to a next task**
**B3**	**Regains focus/focuses again on an assigned task when interrupted** (e.g. classroom announcement, visitor drops in)	**Regains focus/focuses again on an assigned task when interrupted** (e.g., family member asks an unrelated question or needs brief assistance)
B4	**Ignores a preferred, fun activity** (e.g., talking with friends) **when asked to complete an assigned task** (e.g., distribute classroom materials)	**Ignores a preferred, fun activity** (e.g., playing) **when asked to complete an assigned task** (e.g., cleaning room)
B5	**Answers questions or tells a story without getting distracted by small details and/or losing train of thought**	**Answers questions or tells a story without getting distracted by small details and/or losing train of thought**

C. Inhibitory Control - Response Inhibition

C1	**Waits turn to speak** (e.g., does not interrupt teacher or peers, does not blurt out answers, thinks before speaking)	**Waits turn to speak** (e.g., does not interrupt others, does not blurt out answers, thinks before speaking)
**C2**	**Stops an undesirable behavior when asked** (e.g., stops talking with friends when teacher asks during class instruction, stops misbehaving in class)	**Stops an undesirable behavior when asked** (e.g., stops fighting with sibling when asked by parent, stops misbehaving in the household)
C3	**Stops playing or doing something fun when asked** (e.g., stops playing to come back to class, stops socializing with friends)	**Stops playing or doing something fun when asked** (e.g., stops playing with friends when parent asks, stops watching TV when parent asks)
**C4**	**Waits for their turn to act** (e.g., waits patiently in line for recess or when sharing materials with peers)	**Waits for their turn to act** (e.g., waits patiently in line at a grocery store or when sharing a toy with sibling)
C5	**Stops and thinks before acting** (e.g., considers consequences before deciding)	**Stops and thinks before acting** (e.g., does not run out in front of a car, does not grab things they shouldn't)

D. Working Memory

Item No.	Teacher Report	Parent Report
**D1**	**Completes activities that require multiple steps** (e.g., assignment with math calculations, writing composition, art project, dance choreography)	**Completes activities that require multiple steps** (e.g., fetching multiple things in home or store/market, setting the table, cleaning a room by putting several things away)
**D2**	**Completes activities that require remembering lengthy instructions** (e.g., multi-step math problem, science experiment, writing assignment, or classroom chore)	**Completes activities that require remembering lengthy instructions** (e.g., rules of a game, household chores, cooking recipe)
D3	**Communicates in an organized way** (e.g., retells the correct sequence of the events, explains how a game works so it is easy to understand)	**Communicates in an organized way** (e.g., retells the correct sequence of the events, explains how a game works so it is easy to understand)
D4	**Completes age- or grade-appropriate numerical mental calculation** (e.g., math calculations without writing them down, counting backwards)	**Completes age-appropriate numerical mental calculation** (e.g., adds prices of goods, calculates time, how much they are older than someone else)
D5	**Remembers and considers all options when making a choice** (e.g., remembers different activity options before choosing one, remembers different snack options before choosing which one they want to eat)	**Remembers and considers all options when making a choice** (e.g., what to do when they get stuck on a household task or face a problem, food options)
D6	**Remembers physical directions, patterns, and where things are** (e.g., quickly learns seating arrangements or school layout)	**Remembers physical directions, patterns, and where things are** (e.g., knows walking routes, finds things in a grocery store, good at searching for a lost item or playing hide-and-seek)

E. Cognitive Flexibility

Item No.	Teacher Report	Parent Report
**E1**	**Comes up with new ways for solving challenges** (e.g. repurposes materials or toys, comes up with a new way to build a tower or a fort after it collapses, solves a math problem in a new way)	**Comes up with new ways for solving challenges** (e.g., repurposes materials or toys, comes up with a new way to build a tower or fort after it collapses, solves a puzzle in a new way, comes up with a new strategy to complete a chore)
E2	**Expresses themselves in a new way when not understood by others** (e.g., explains answer to a problem in different ways during group work)	**Expresses themselves in a new way when not understood by others** (e.g., tries different ways to communicate their needs and wants)
**E3**	**Adjusts when plans/schedules change** (e.g., follows along when class schedule changes, does not complain when teacher does a different activity than planned)	**Adjusts when plans/schedules change** (e.g., follows along when daily schedule changes, does not complain when unexpected sickness leads to canceling a family vacation)
E4	**Understands conflicting perspectives/ideas** (e.g., acknowledges a different point of view, accepts different ways of doing things, can see a situation from someone else's perspective)	**Understands conflicting perspectives/ideas** (e.g., acknowledges a different point of view, accepts different ways of doing things, can see a situation from someone else's perspective)
E5	**Connects ideas/experiences to other ideas/experiences** (e.g., identifies similarities or differences between objects, concepts, or people; shares a personal experience that's related to an academic topic or book; uses strategies learned at home to complete activities at school)	**Connects ideas/experiences to other ideas/experiences** (e.g., identifies connections between books/movies and lived experiences, uses strategies learned at school to complete activities at home)
E6	**Adapts behavior based on what is required by the situation** (e.g., switches characters/roles while playing a game, changes behavior based on class time or break time)	**Adapts behavior based on what is required by the situation** (e.g., switches characters/roles while playing a game, changes behavior based on playtime or study time)

F. Planning & Organization

**Item No.**	**Teacher Report**	**Parent Report**
F1	**Identifies and gathers necessary materials before an activity** (e.g., finds appropriate coat, shoes, hat before going outside; gathers paper, markers, and scissors before an art activity)	**Identifies and gathers necessary materials before an activity** (e.g., finds appropriate coat, shoes, hat before going outside; gathers lunch box, homework, and backpack before going to school)
F2	**Creates a strategy or plan for completing a task** (e.g., talks through how he/she will solve a class assignment, puzzle)	**Creates a strategy or plan for completing a task** (e.g., chore, homework, puzzle)
**F3**	**Finishes tasks on time** (e.g., turns homework on time, meets class deadlines)	**Finishes tasks on time** (e.g., finishes homework on time and not at the last minute)
F4	**Checks for mistakes before completing a task** (e.g., reviews and corrects errors on class assignments)	**Checks for mistakes before completing a task** (e.g., reviews and corrects errors on class assignments)
**F5**	**Begins plans or routines without prompting** (e.g., takes an initiative to clean up, starts daily routine classroom activities on their own)	**Begins plans or routines without prompting** (e.g., takes an initiative to help a family member, starts daily household chores on their own)

Table S2

EFFORT Assessor Report

Item No.		4	3	2	1
1	**Pays attention during task instructions and demonstrations**	attention does not waver, listens carefully and watches all task demonstrations with appropriate eye gaze and/or body orientation	attention drifts once or twice, but refocuses on their own	attention occasionally drifts, but is responsive to a single prompt	attention frequently drifts and the assessor provides repeated prompts and/or extra support (e.g., repeating instructions, changing seating position)
2	**Finishes an assessment task that is repetitive**	sustains concentration, not distracted and finishes all tasks on their own	distracted once or twice but generally persistent, does not require a prompt to finish	occasionally distracted, but is responsive to a single prompt from assessor to finish	frequently distracted, requires repeated prompts and/or extra support to finish a task (e.g., praising effort)
3	**Remains focused and continues to work during a lengthy task**	remains focused and continues to work during entire task, engagement does not waver	focused during most of the task; may stop working on task once or twice, but re-focuses on their own	focus occasionally drifts during a task; stops working on task occasionally, but responsive to a single prompt to re-focus	focus frequently drifts and frequently stops work on a task, and requires repeated prompts and/or extra support to refocus
4	**Persists on a difficult task by trying to figure things out, asking questions, and not quitting**	remains focused and persists during all aspects of challenging tasks, engagement does not waver	mostly focused during challenging tasks, may get disengaged once or twice, but persists on their own	focus occasionally drifts during challenging tasks, persists with a single encouragement to stay engaged and persist.	focus frequently drifts during challenging tasks and needs several encouragements (e.g., you are almost done, try your best)

Item No.		4	3	2	1
5	**Ignores irrelevant activity/noise when working on an assigned task**	does not become distracted by surrounding noise; able to resist looking around	may get distracted once or twice by surrounding noise (e.g., looks away once from the task) but refocuses attention on their own	occasionally becomes distracted (e.g., looks around for a short period), but returns to task with a single prompt to ignore irrelevant activity/noise	often distracted, spends significant time looking away from the task, requires multiple prompts and/or extra support (e.g., reducing external distractions)
6	**Completes an assigned task without rushing before moving to the next task**	finishes a current task completely and does not inquire about other activities or materials while doing it	finishes a current task, but may rush towards the end to get to the next task quicker	occasionally will ask to do a new task before completing an old one, but is responsive to a single prompt to slow down	frequently will ask to do a new task before completing an old one; keeps asking about other activities/task
7	**Waits turn to speak**	always waits for the assessor to finish asking a question or provide instructions	once or twice interrupts to ask a question, but generally shows good self-control	occasionally interrupts to ask a question, share thoughts, and/or offers answers, but is responsive to a single reminder to wait	interrupts multiple times and requires several reminders about waiting for assessor to finish talking

Item No.		4	3	2	1
**8**	**Stops an undesirable behavior** (e.g., touches materials, wiggles in chair) **when asked**	does not display undesirable behavior (i.e., displays self-control with all presented toys, stimuli, and materials).	displays undesirable behavior once or twice (e.g., reaches for materials out of turn), but generally shows good self-control	occasionally displays undesirable behaviors, but is responsive to a single prompt to stop	frequently displays undesirable behavior and needs multiple reminders to stop
**9**	**Patiently waits for new task to begin**	always waits patiently for new tasks to begin	once or twice displays fleeting impatience	occasionally can't wait to start the next task, but is responsive to a single prompt to wait	child cannot wait for the next task most of the time and it is difficult to start the next task
**10**	**Checks for mistakes before completing a task**	always checks for mistakes before completing a task and corrects mistakes on their own	may miss 1-2 mistakes before completing a task, but generally checks their work	child occasionally makes mistakes without noticing and need a reminder from assessor about rules and to notice mistakes	child frequently makes mistakes without noticing and needs repeated reminders from assessor about rules and to notice mistakes

Table S3

Number of Observations for Study Samples

Site	Total Children	Caregiver Report	Teacher Report	Assessor Report	Direct Assessment	Caregiver and Teacher Report	Caregiver and Direct Assessment	Teacher and Direct Assessment	Assessor and Direct Assessment
Full Sample	1738	1262	892	1193	1243	426	776	682	1156
Argentina	93	49	88	46	81	44	40	66	39
Australia	275	218	227	261	269	179	213	224	260
Bangladesh	448	448	58	448	421	58	421	54	421
Haiti	353	NA	353	338	351	NA	NA	338	338
South Africa	104	104	NA	100	102	NA	102	NA	98
Sri Lanka	177	155	166	NA	NA	145	NA	NA	NA
United States	288	288	NA	NA	NA	NA	NA	NA	NA

Note. Numbers of observations for each sample based on non-missing data. NA indicates the data was not applicable for the sample.

Table S4

Site Level Study Characteristics

**Site**	**Child Sample**	**Age** *M* (*SD*)	**% Female**	**Sample Strategy**	**Region**	**Language**	**% CG Secondary Education[1]**	**EFFORT Reporter**	**Direct Assessments**
Argentina	93	4 to 5 years *5.36 (0.56)*	47%	Purposeful	Buenos Aires	Rioplatanese Spanish	100 %	C, T, A	HF, Memory Game
Australia	275	3 to 5 years *4.68 (0.51)*	43%	Ongoing study	New South Wales	English	94.8 %	C, T, A	Go/No-Go, HTKS
Bangladesh	448	5 to 11 years *7.97 (1.99)*	50%	Purposeful	Rangpur and Magura Districts	Bangla	20.3 %	C, T, A	HF, Memory Game
Haiti	353	4 to 8 years *5.29 (0.71)*	51%	Ongoing study	Nord and Artibonite Districts	Haitian Kreyol	6.2 %	T, A	IDELA HTKS, Forward digit span
South Africa	104	3 to 11 years *7.16 (2.33)*	50%	Ongoing study	Cape Town	English	84.0 %	C, A	Forward and backward digit span, HF
Sri Lanka	177	4 to 7 years *6.61 (0.59)*	45 %	Purposeful	Kurunegala District	Tamil and Sinhala	96.7 %	C, T	NA
United States	288	4 to 11 years *7.92 (2.08)*	47%	Convenience	NA	English	NA	C	NA

Note. Table describes site level information for each study sample. The EFFORT Reporter column indicates which reporters were administered EFFORT at the site. C is for caregiver, T is for teacher, and A is for assessor. The Direct Assessments column indicates the measures at each site. HF is for Hearts and Flowers. HTKS is for Head-Toes-Knees-Shoulders. The digit span is a short-term and working memory measure. NA indicates not applicable for the site. [1] Education was not available for all caregivers, we report % of available responses.

Figures

Figure S1

Teacher Item Histograms

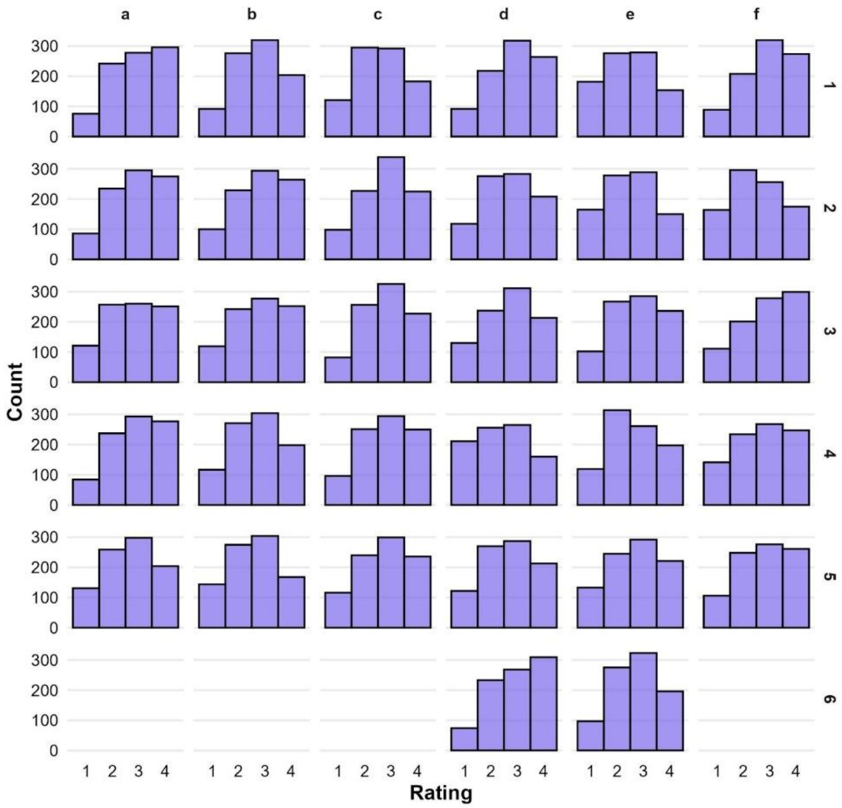

Figure S2

Caregiver Item Histograms

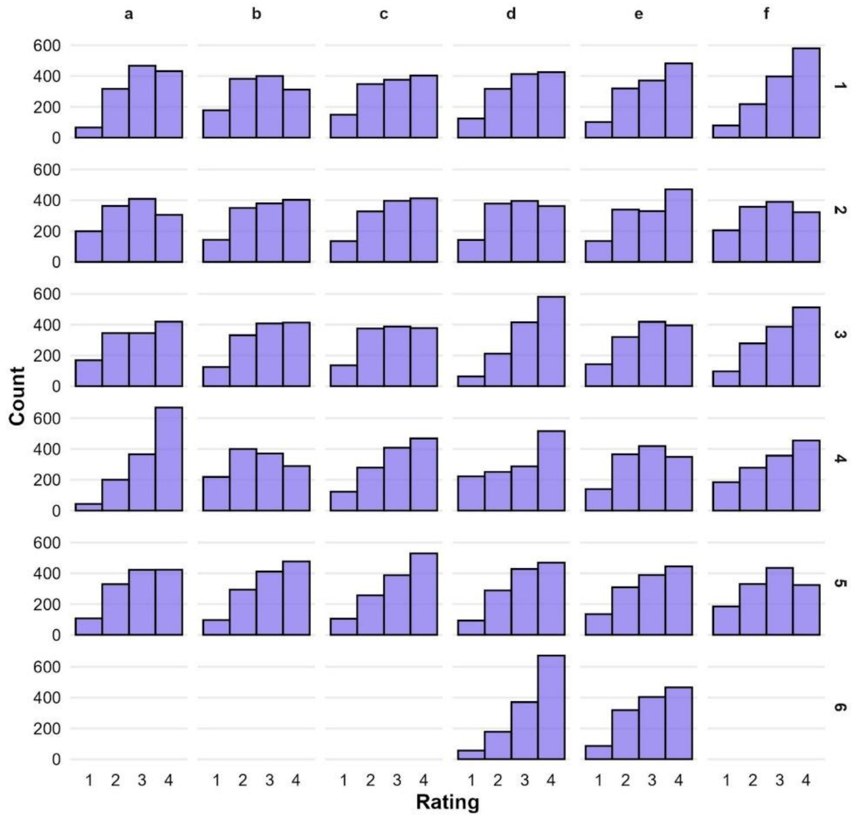

Figure S3

Assessor Item Histograms

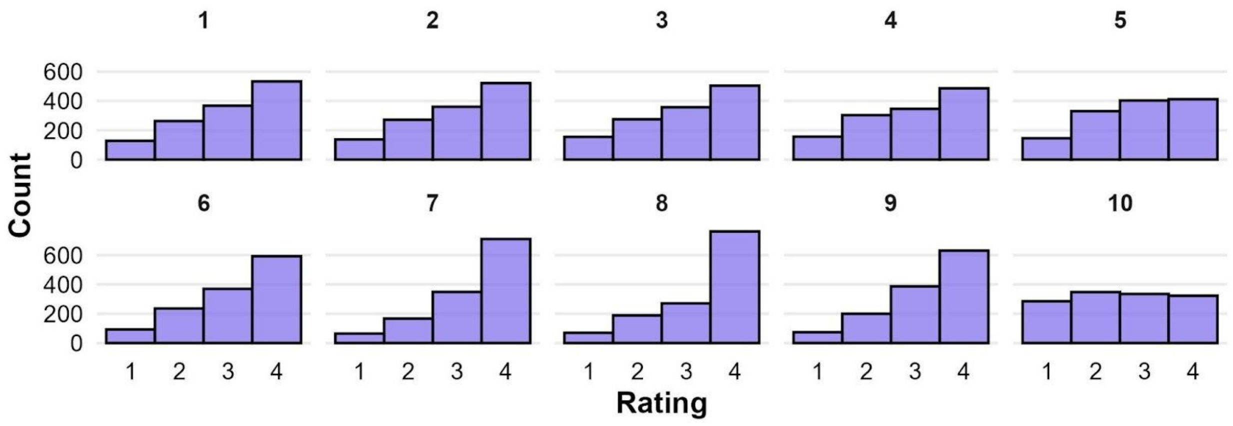

Text S1

Translation and Adaptation of EFFORT

The team created a list of item-level translation and adaptation guidelines shared with each study site and also accessible on OSF.

Each site adapted the EFFORT items to the local context by adapting the examples to be contextually relevant and translating into the local language. The full site-specific items are available on OSF. Below are the specific steps taken at each site for translation and adaptation to guide similar processes in future studies.

Argentina. The EFFORT caregiver, teacher, and assessor items were translated and adapted into Rioplatense Spanish, the Spanish dialect spoken in Buenos Aires. This process was conducted by five members of the research group, including researchers and fellows with backgrounds in psychology and anthropology, as well as the field coordinator. Two team members independently translated each item from English into Spanish, after which the remaining three members reviewed the translations and provided feedback. Through this iterative review process, the team developed a provisional consensus version of the survey. This version was then discussed in two meetings with the four assessors who comprised the field team prior to implementation. The final version used in the pilot study emerged from this final round of discussion. While most examples closely followed the original English version, some examples were added or modified to better reflect daily family life in the local context (e.g., adding references to handwriting practice or family outings). To support respondents' comprehension of the response scale, a visual Likert-scale aid was used.

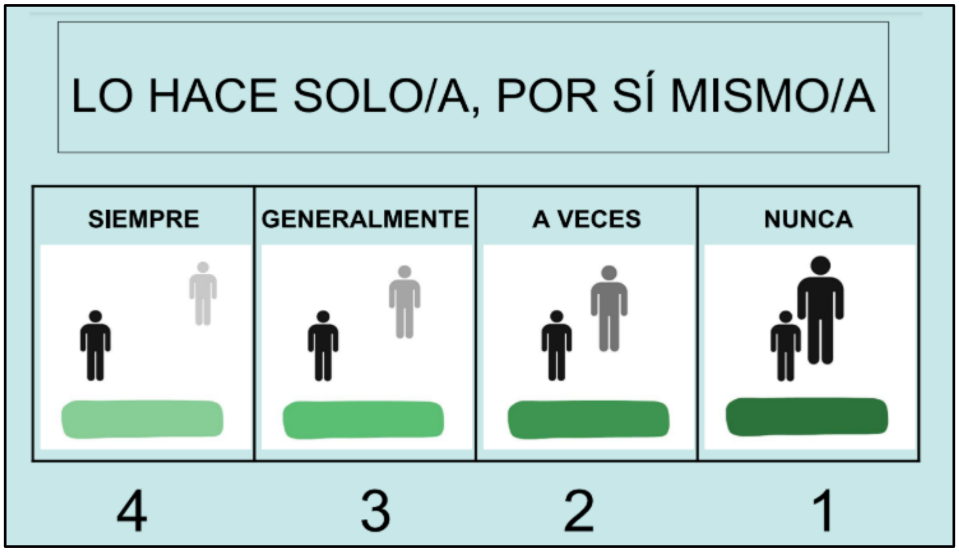

Figure S4. Image used to facilitate family responses during EFFORT administration. Note: "Los hace solo/a, por si mismo/a": He/she does it alone, by himself/herself; "Siempre": Always; "Generalmente": Frequently; "A veces": Sometimes; "Nunca": Never.

Australia. No language translation was required, as all EFFORT caregiver, teacher, and assessor items were administered in English. However, the research team conducted a systematic review of the surveys to ensure terminological alignment and contextual relevance to the Australian early childhood education and care (ECEC) sector. This review was undertaken by a multidisciplinary team with expertise in child development, executive functions, and ECEC practice, including educators. As a result, minor adaptations were made to the educator survey, including changes in terminology (e.g., replacing the term “teacher” with “educator”), removal of examples that were uncommon in Australian ECEC settings (e.g., ‘assignment with math calculations’), and the addition of analogous examples more familiar to local educators (e.g., waiting patiently in line or following a building plan). These revisions were intended to improve clarity and relevance without altering item constructs.

Bangladesh. The EFFORT caregiver, teacher, and assessor items were translated from English into Bangla and culturally adapted through a collaborative process involving the principal investigator and a local field researcher. Following the initial translation and adaptation, the items were reviewed by a second, senior local researcher to assess clarity, conceptual equivalence, and contextual relevance. A back-translation into English was then conducted to verify that the Bangla items reflected the same underlying constructs as the original English version. Minor adaptations were made to simplify language and improve comprehension, including the use of simpler verbs (e.g., “tells” instead of “communicates”) and the substitution of locally familiar examples (e.g., “builds a house with dirt or mud” in place of “builds a fort”). In addition, some wording adjustments were required in the assessor report because several English distinctions (e.g., “pays attention,” “focused,” and “engaged”) map onto a single term in Bangla.

Haiti. The EFFORT teacher and assessor items were translated from English into Haitian Kreyol using a multi-step process. First, the English versions were reviewed by the US-based research and program team to assess relevance and feasibility, after which no changes were deemed necessary. The surveys were then translated into Haitian Kreyol by a professional translator. The translated versions were subsequently reviewed by the Haiti-based research and program team, which included former preschool teachers serving as coaches. This team assessed the translations for clarity, relevance, and feasibility and introduced minor adaptations. These included adding a short instruction section to the teacher survey to clarify the purpose of the data collection, survey completion procedures, and response options, as well as making small wording changes to better align examples with common preschool routines (e.g., using “waits in line” instead of “waits for their turn”; changing “sharing circle” to “morning meetings”, using “build a house” rather than “build a fort”, and using “reuse toys in different ways,” rather than “repurpose toys”). Because no major content changes were made, a professional back-translation was not conducted; instead, a bilingual team member reviewed the final Kreyol version to confirm conceptual equivalence with the English items.

South Africa. The EFFORT caregiver and assessor items were administered in English, following a review by the locally based research team to assess relevance and feasibility. Minor edits were made to adapt item examples to better reflect local daily activities and contexts familiar to participants. For instance, examples referencing classroom-based academic tasks were replaced with activities such as building with blocks, coloring, playing games, or completing household chores. Although the survey was not formally translated into isiXhosa, the language most commonly spoken at the research site, two isiXhosa-fluent research assistants reviewed all items and examples and reached agreement on how to explain the questions orally in isiXhosa when needed during administration.

Sri Lanka. The EFFORT caregiver and teacher items were first translated from English into Sinhala and Tamil by a group of certified translators. The translated items, along with their instructions, were then reviewed through an iterative feedback cycle involving local educators, researchers, and the team of trained assessors. This review focused on ensuring accuracy, readability, and cultural relevance, with particular attention to the use of locally resonant terminology to describe executive function–related behaviors. The examples included in the EFFORT survey were largely retained, as they were judged to reflect commonly observed behaviors in the local context.
